# The effect of intrinsic and extrinsic motivation on memory formation: insight from behavioral and imaging study

**DOI:** 10.1007/s00429-020-02074-x

**Published:** 2020-04-29

**Authors:** Hongxia Duan, Guillén Fernández, Eelco van Dongen, Nils Kohn

**Affiliations:** 1 Cognitive Neuroscience Department, Donders Institute for Brain, Cognition and Behavior, Radboud University Medical Center, Kapittelweg 29, 6525 EN Nijmegen, The Netherlands; 2 Center for Brain Disorder and Cognitive Science, Shenzhen Key Laboratory of Affective and Social Cognitive Science, Shenzhen University, Shenzhen, 518060 China

**Keywords:** Curiosity, fMRI, Motivation, Reward

## Abstract

Motivation can be generated intrinsically or extrinsically, and both kinds of motivation show similar facilitatory effects on memory. However, effects of extrinsic and intrinsic motivation on memory formation have not been studied in combination and thus, it is unknown whether they interact and how such interplay is neurally implemented. In the present study, both extrinsic monetary reward and intrinsic curiosity enhanced memory performance, without evidence for an interaction. Functional magnetic resonance imaging revealed that curiosity-driven activity in the ventral striatal reward network appears to work cooperatively with the fronto-parietal attention network, while enhancing memory formation. In contrast, the monetary reward-modulated subsequent memory effect revealed deactivation in parietal midline regions. Thus, curiosity might enhance memory performance by allocation of attentional resources and reward-related processes; while, monetary reward does so by suppression of task-irrelevant processing.

**Electronic supplementary material:**

The online version of this article (10.1007/s00429-020-02074-x) contains supplementary material, which is available to authorized users.

## Introduction

The challenge of how to stimulate students’ motivation to learn has always been one of the central topics of education. Should children be rewarded to achieve a high grade or be motivated from within themselves to seek out unknown information? The former relies on extrinsic motivation, which refers to doing things for the sake of obtaining an externally tangible reward such as good grades, praise, or money. The latter, intrinsic motivation, refers to doing things following an internal desire such as curiosity. Behaviorally, both intrinsic and extrinsic motivation can facilitate memory formation and modify goal-directed behavior (Loewenstein 1994; Ryan and Deci 2000).

Dopaminergic midbrain regions (e.g., substantia nigra/ventral tegmental area: SN/VTA) and their striatal projections, especially to the nucleus accumbens (NAcc) are thought to support reward anticipation (Zellner and Ranaldi 2010) and declarative memory formation depends on the medial temporal lobe (MTL) with the hippocampus at its core (Squire et al. 2004). Evidence from human and animal studies supports the view that the MTL memory system and the striatal reward system comprise a functional loop (Lisman and Grace 2005; Rossato et al. 2009) in which the VTA and NAcc modulate medial temporal activity underlying successful memory formation (Adcock et al. 2006; Bunzeck et al. 2012; Wittmann et al. 2005).

Learning can also occur in a self-motivated manner, driven by epistemic curiosity, the “motivation to know”, to fill an information gap (Loewenstein 1994) or an intrinsic desire to reduce uncertainty (Kidd and Hayden 2015). Curiosity evoked as a form of “cognitively induced deprivation that arises from the perception of a gap in knowledge and understanding” is regarded as an unpleasant state, and relief of this condition, through access to new information that reduces this state, is rewarding and promotes learning (Berlyne 1954; Loewenstein 1994). Compared to extrinsic reward, less is known about how intrinsic curiosity affects the neural processes underlying memory formation. An initial study by Kang et al. (2009) found that curiosity levels to trivia questions were correlated with increased activation in inferior frontal gyrus, parahippocampal gyrus and caudate nucleus, and curiosity was also correlated with increased activation in these regions when answers were revealed, but participant guessed incorrectly (i.e., had less prior knowledge) (Kang et al. 2009). Curiosity not only interacts with prior knowledge, but also enhances new learning. In a study by Gruber et al. (2014), curiosity enhanced memory performance in a one-day delay test, and this curiosity-driven learning benefit was associated with activity in the SN/VTA and hippocampus as well as their neural interaction (Gruber et al. 2014).

A common interpretation is that curiosity, as anticipation of rewarding information, enhances memory by sharing common neural mechanism with extrinsic motivation, but this proposal has not been tested directly. However, previous behavioral studies, have shown that extrinsic motivation can also undermine intrinsic motivation (the “undermining effect”) (Deci et al. 1999). This undermining effect was partially supported by a behavioral study in which monetary reward improved memory performance but only to uninteresting questions and had no effect on interesting questions in a one-week delayed surprise memory test. However, in that study, the level of interest in the questions was rated by 20 independent raters rather than the participants of the memory task themselves (Murayama and Kuhbandner 2011), calling into question the degree to which this level reflected the intrinsic motivation of the participants or some other factor of interestingness. Murayama et al. (2010) investigated the neural mechanism of this undermining effect in which performance-based monetary reward impaired intrinsic motivation as indicated by less amount of voluntary engagements in the free-choice task. They found that activity in value-related striatum region and cognitive control-related lateral prefrontal cortex decreased with this behavioral undermining effect. In contrast to the undermining effect, a recent meta-analysis demonstrated that the effect of intrinsic motivation on performance was larger in the presence of extrinsic incentives (“additive effect”) (Cerasoli et al. 2014). Thus, there is conflicting evidence on how intrinsic and extrinsic motivation interact when modulating memory, and the neural underpinnings of this interaction are largely unknown.

**Fig. 1:**
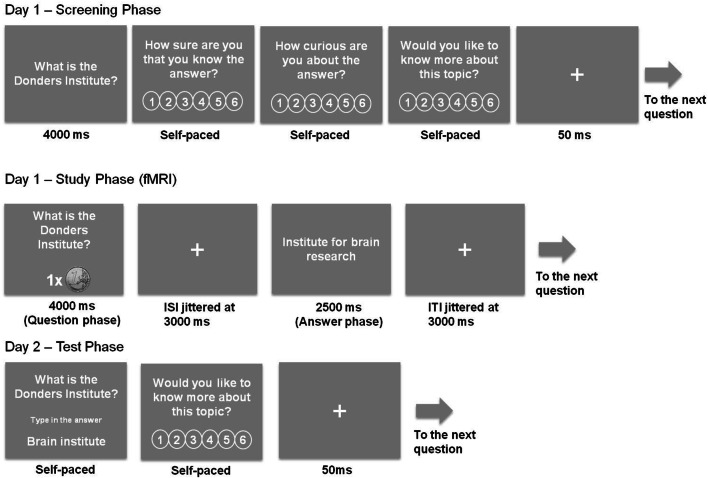
Experimental procedure: Screening and study phase lasted approximately 4.5 h on day 1 and the test phase 1.5 h on the next day (mean study-test delay 20.5 h, SD: 0.4 h)

Here, we used a modified version of the trivia question task (Fig. 1) (Gruber et al. 2014; Kang et al. 2009) to investigate the behavioral and neural interaction between intrinsic (curiosity) and extrinsic motivation (monetary reward) and how they modulate memory formation. Given that both monetary reward and curiosity can independently enhance long-term memory, we predicted that their presence would be associated with neural interaction between the reward system (ventral striatum) and the MTL memory system. We hypothesized that monetary reward would reduce interest/engagement for high curiosity questions and, therefore, impair memory performance with high rather than low curiosity ratings, which is in line with the undermining effect (Deci et al. 1999). Alternatively, there could be an additive effect of monetary reward and curiosity on memory, i.e., monetary reward and curiosity would enhance memory performance independent of each other and, thus, would be associated with different neural correlates.

## Materials and methods

### Participants

Thirty-five healthy right-handed young participants (mean age 22.9 years, standard deviation (SD): 3.13 years; 22 females) were recruited for this study. Prior to participation, volunteers were screened for the following exclusion criteria: non-native Dutch speaker, dyslexia, (history of) neurologic or psychiatric disorders, (history of) somatic diseases with a possible influence on brain structure or function, and conventional MRI contraindications. Two participants were excluded from fMRI analyses due to missing data. In total, there was 33 participants with six participants who had only fMRI data available for half of the sessions due to technical problems with the eye-tracking system in the final data analysis. All participants were paid 64 Euros plus a bonus dependent on their memory performance (see below for details). They received a complete description of the study after which the written informed consent was obtained. All participants were fully informed on the nature of the study after completion. This study was approved by the local ethics committee (CMO Region Arnhem-Nijmegen, the Netherlands) and was conducted in accordance with the Declaration of Helsinki.

### Task procedures

Participants underwent a three-stage paradigm with (1) a screening phase in which they classified trivia questions according to individual prior knowledge and curiosity, (2) a study phase in which they learned a series of 270 individually selected trivia answers while being scanned and (3) a test phase on the next day during which their memory for the studied trivia answers was tested. To manipulate intrinsic and extrinsic motivation individually, trivia information was binned during the study phase according to a full factorial design with two factors, each having three levels: Curiosity (Low/Middle/High) and Monetary reward (None/Low/High = 0/1/3 Euros) using data obtained in the screening phase from each particular participant. Participants were told that their memory for trivia answers would be tested on the next day and that 16 random trivia questions would be selected and rewarded. For each of these 16 trivia items that they correctly remembered at test, they received the associated monetary reward (0/1/3 Euros) as a bonus on top of the standard participation fee.

#### Screening phase

To create participant-specific stimulus sets, each participant rated 770 trivia questions on 6-point scales for (a) their confidence in whether they already knew the answer to the trivia question and (b) their curiosity for getting to know this answer (c) their prospective curiosity about the topic (see Fig. 1). Trivia questions were randomly selected from different current editions of the game Trivial Pursuit (Dutch Genus edition 2005; Dutch master edition 2011), using the categories history, science and geography. Trivia questions were presented one by one and the rating of the questions was self-paced (see Fig. 1). If a participant did not know the answer to a trivia question (≤ 4 rating of knowledge level, see Fig. S1 in Supplementary Material), it was considered further and grouped into one of the three curiosity bins: low curiosity (1–2 rating on curiosity), middle curiosity (3–4 rating on curiosity) and high curiosity (5–6 rating on curiosity). Based on this procedure, we created participant-specific stimulus sets, each with 270 trivia questions (90 questions per curiosity bin) that were not yet known to the participant and which were subsequently used for the study phase. Participants would not be invited to the study and test phase if there were too few curiosity trials for each bin (at least 72 each bin; only one participant was included with only 264 trials instead of the full 270). Excluded participants were paid for the screening phase. To make sure that participants would not get too tired, there were breaks built into the screening phase at different moments. These breaks would last at least 1 min, after which participants could determine when to continue. In total, the screening phase took about 3.5 h including enforced breaks.

#### Study phase

After participants finished the screening phase, they continued with the study phase in an MRI scanner (see below for details). The 270 trivia questions selected during the screening phase were presented along with the associated answers (i.e., the corresponding answers, see Fig. 1). Each trial started with the presentation of a trivia question for 4 s combined with the monetary reward level (0/1/3 Euros) presented below the question. Following a fixation cross ISI (3–5 s), the associated trivia answer was presented for 2.5 s. After another fixation cross ISI (3–5 s), the next trial started. The study phase took approximately 1.5-h in total and was divided into two runs, separated by an anatomical scan (see below for details).

#### Test phase

On average 20.5 h after the start of the study phase (SD: 0.4 h), the recall test for the 270 studied trivia questions was administered. All trivia questions presented at study were repeated one by one in the same order as they were studied, and participants were instructed to retrieve the correct answer and type it on a keyboard without time limit. Afterwards, participants rated their prospective curiosity about the topic on a 6-point scale (see Fig. 1).

After participants finished the recall task, their answers were rated by two independent raters by comparing the input from the participants with the correct answers to determine memory performance. Then, participants received a bonus based on the performance of the randomly selected 16 trials as described above. In the end, participants were debriefed.

### Statistical analysis

#### Behavioral analyses

Behavioral analyses were conducted with SPSS 21 (IBM Corp., Armonk, NY, USA). A full factorial design was implemented for memory accuracy in a generalized estimating equations (GEE) procedure, with the factors of monetary reward and curiosity, controlled for the level of prior knowledge (1–4) per trivia question. GEE is a generalization of generalized linear model approaches that considers within-subject correlations and also allows explicit specification of link functions. Models were the within-subject factors reward and curiosity, as well as their interaction. Knowledge level was included as a covariate to control for effects of pre-existing knowledge on memory, which would most likely also influence learning. A logit link function was implemented and an autoregressive model of the first order was used to initialize the working correlation matrix. Pairwise comparisons (conducted after significant interactions) were corrected for multiple comparisons by sequential Sidak.

Additionally, to test if the levels of curiosity and reward have an influence on prospective curiosity, we first calculated the change in prospective curiosity from screening to recall. A difference score was calculated by subtracting prospective curiosity rating (“would you like to know more about the topic”) at the *Test* phase to prospective curiosity rating at the *Screening* phase. Therefore, the smaller the difference score, the more the prospective curiosity drops after test during which curiosity for the answer to the trivia question is satisfied. Next, a full factorial design was implemented for the difference score in a GEE procedure, with the factors of monetary reward and curiosity, controlled for the level of prior knowledge (1–4) per trivia question.

### fMRI data acquisition

A 3T Siemens Skyra scanner (Siemens; Erlangen, Germany) was used at the Donders Institute in Nijmegen, the Netherlands. A multiband-4 Echo-Planar Imaging sequence was used to acquire the whole brain T2*-weighted images (multi-band factor = 4; repetition time (TR) = 1.7 s, echo time (TE) = 35 ms, 2.4 mm isotropic, flip angle = 75°, slice thickness = 2 mm, field of view (FOV) = 210 mm, interleaved acquisition, Orientation AC-PC). A 3D magnetization-prepared rapid gradient-echo (MPRAGE) anatomical T1-weighted image (192 slices, 1.0 mm isotropic, TR = 2300 ms, TE = 3.03 ms, flip angle = 8°, slice thickness = 1 mm, FOV = 256 mm, sequential acquisition, sagittal orientation) with whole-brain coverage was acquired for anatomical normalization. Stimuli were projected onto a mirror attached to the head coil. During scanning, the participant’s eyes were monitored by the experimenter via an eye-tracker system (iView X version 2.8.26) to make sure that participant looked at all stimuli.

### fMRI preprocessing

The functional and anatomical images were preprocessed and analyzed using FSL software (FMRIB, University of Oxford, UK; www.fmrib.ox.ac.uk/fsl; Jenkinson et al. 2012). The first three volumes of each functional time series were discarded to disregard magnetization effects and initial transient signal changes. For each participant, we first visually inspect the raw data and outputs of each preprocessing step to find the obvious artefacts or abnormal image. Functional images were spatially smoothed with a 5-mm full-width at half-maximum Gaussian kernel to reduce inter-subject variability. The three-dimensional movement correction by MCFLIRT was first applied (Jenkinson et al. 2002). We further visually explored subject-wise registration results as well as group masks generated by FSL for the group-level analyses. Next, ICA-AROMA was used to automatically remove motion artifacts and other noise components. This tool uses linear regression of ICA components, identifies components as noise and (non-aggressively) regresses out the time courses of these components. This ICA-based denoising has been demonstrated to be effective in removing aberrant activation resulting from subject motion and performs superior to multiple other approaches in motion correction (Pruim et al. 2015). Subsequently, a high-pass filter of 100 s was administered. Prior to group analyses, individual functional images were normalized to MNI space in a two-step procedure combining linear and non-linear registration (Andersson et al. 2007; Jenkinson et al. 2002).

### fMRI analyses

General linear models (GLMs) were estimated on preprocessed and denoised 4D files of each participant and session. There were three regressors for all of the trials: one regressor codes trials regardless of condition, the second is a parametrically modulated regressor based on the reward level and the third is a parametrically modulated regressor based on the curiosity level. Additionally, one regressor is created for the trials in which the highest reward and curiosity levels were combined. In this way, the specific effects of the combination of high reward and high curiosity would be detectable. These separate regressors were created for trivia question presentation and trivia answer presentation (and separately for remembered and forgotten trials). The ISI was explicitly modeled in the analysis, because participants most likely engage in memory retrieval or search processes during this period. Thereby, not modeling this phase would potentially decrease model fit. The duration of eye-closure time per trial (as measured with the eye-tracking system) was included as an additional nuisance regressor. Furthermore, we calculated the variance inflation factor (VIF), a measure of multicollinearity, for the regressors of interest. To obtain the mean VIF for each regressor of interest (during the question phase: Curiosity_rmb & fgt, Reward_rmb & fgt, and Curiosity X Reward_rmb & fgt; the same regressors of interest for during the answer phase; ISI), we extracted the respective regressors from the design matrix for first and second runs separately (temporal derivatives of the regressors were not included). The VIF was computed for each of these regressors and then averaged across two runs. The maximum VIF is 8.6 and the mean VIF for all the regressors is 4.93. Therefore, all VIFs are within a reasonable range for assessing multicollinearity, in which a value over 10 is considered problematic (Stevens 1996). This finding indicates that including ISI as regressors in the model did not cause over-specification of the GLM model. A box-car regressor convolved with a canonical hemodynamic response function corresponding to the question and answer presentation was modulated with reward and curiosity level, respectively. To establish the brain activation pattern of motivation for fully processed trials, these analyses were conducted on trivia questions for which the correct answers were later remembered.

To test the benefit of motivation on learning, the analyses of interest were conducted on the trivia answer phase (answer shown on screen), since this is the stage when the later tested information is presented. Each trivia answer was classified according to whether it was later correctly recalled or forgotten. This subsequent memory effect was further qualified by the level of curiosity and monetary reward. For the later remembered items, the average trial numbers for Low Curiosity combined with three levels of Monetary Reward (None/Low/High) are 11.49/12.94/13.74; the average trial numbers for Middle curiosity combined with three levels of Monetary Reward (None/Low/High) are 15.31/16.23/17.14; the average trial numbers for High curiosity combined with three levels of Monetary Reward (None/Low/High) are 19.60/20.06/20.37. For the later forgotten items, the average trial numbers for Low Curiosity combined with three levels of Monetary Reward (None/Low/High) are 18.37/16.86/15.94; the average trial numbers for Middle curiosity combined with three levels of Monetary Reward (None/Low/High) are 14.83/13.97/13.14; the average trial numbers for High curiosity combined with three levels of Monetary Reward (None/Low/High) are 10.31/10.0/9.49. We compared subsequently remembered to the subsequently forgotten trials. In short, for each participant, motivation-related brain activation was conducted in the question presentation phase and modeled with two regressors (monetary reward and curiosity) whose magnitudes scaled linearly with curiosity bins and monetary reward levels separately. For the activation predicting later memory of trivia answers, the main analysis was conducted in the answer presentation phase and the curiosity/reward-modulated remembered trials was compared with forgotten trials.

For group statistics, we analyzed these models using the mixed effects approaches FLAME 1 + 2 on the whole brain. All reported z-statistic images were thresholded using clusters determined by *z* > 2.3 and a corrected cluster significance thresholded at *p* < 0.05 for the whole brain (Worsley 2001). Images were overlaid and viewed onto standard brain in MNI space using MRIcroGL (https://www.nitrc.org/projects/mricrogl).

#### Region of interest analysis

In addition to the whole-brain fMRI analyses, we conducted ROI analyses on the NAcc to explore the specific role of mesolimbic regions on reward and curiosity, as well as ROI analyses on the hippocampus to explore its role in memory formation. This selection of NAcc and Hippocampus is inspired by the ROI analyses conducted on the paradigm that we modified for our current study (Gruber et al. 2014). Briefly, the NAcc and the hippocampus demonstrate strong intrinsical connectivity (Kahn and Shohamy 2012) and their connectivity has been proposed to support learning (Lisman and Grace 2005). The bilateral NAcc and bilateral hippocampus were derived from the NAcc mask of the FSL’s Harvard–Oxford Cortical and Subcortical Probabilistic Atlases (Desikan et al. 2006) with a probabilistic threshold = 50% probability.

## Results

### Behavioral analysis

The average memory performance was 54.26% correct (standard deviation: 20.57). There was a positive relationship between prior knowledge level and memory performance (*r* = 0.207, *p* < 0.001). Therefore, we investigated the effect of curiosity and monetary reward on memory performance with prior knowledge level controlled.

**Fig. 2:**
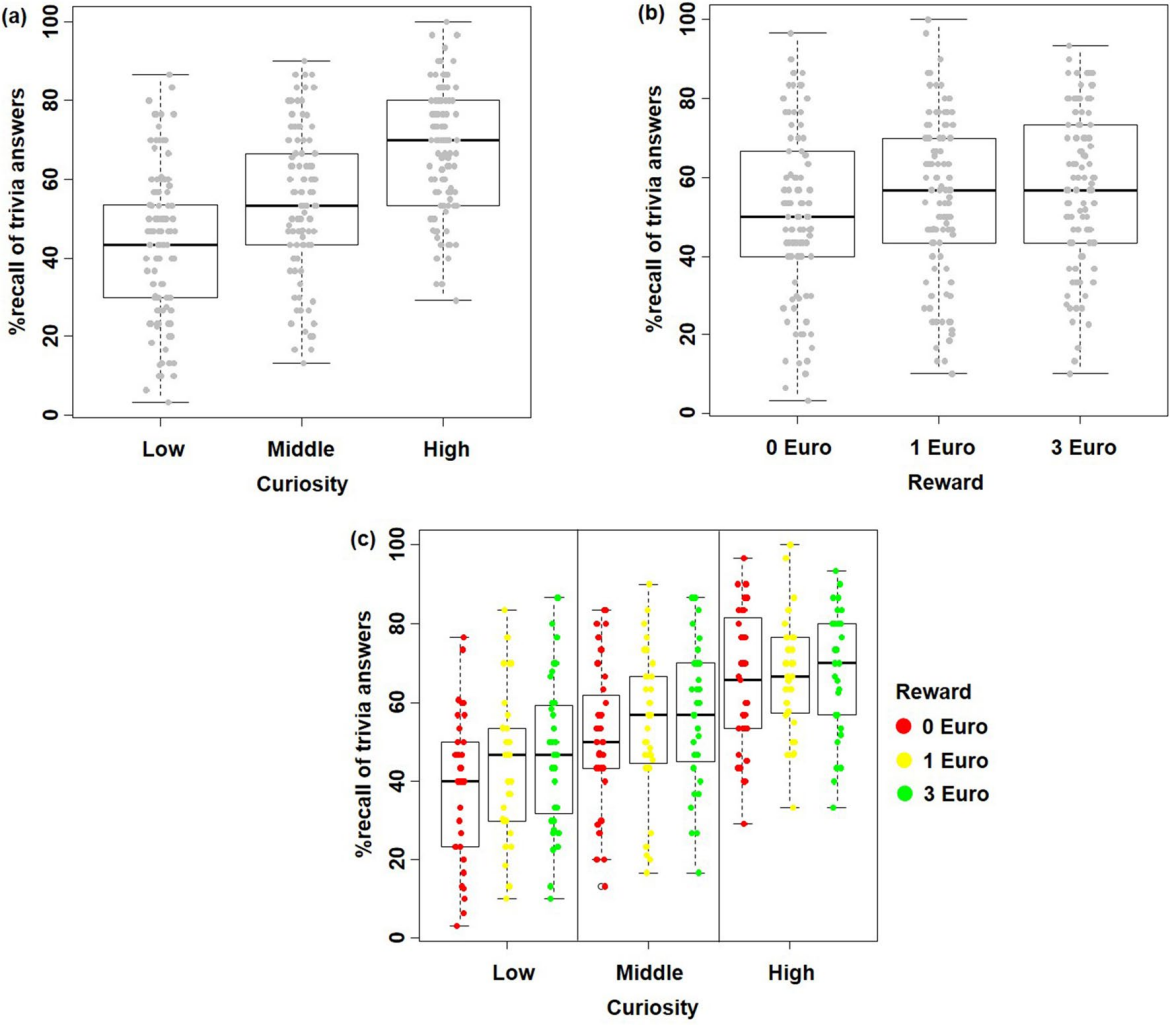
**a** The main effect of Curiosity on prospective curiosity. **b** The main effect of Monetary Reward on prospective curiosity. **c** No significant interaction effect of Curiosity and Monetary Reward on prospective curiosity

Results show an effect of curiosity on memory: the higher the level of curiosity, the better the participants recalled the answer (Curiosity (Low/Middle/High): 43/54/67% correct; Wald χ^2^ = 186.05; *p* < 0.001)), which replicates previous findings (Gruber et al. 2014; Kang et al. 2009). The effect of reward on memory was also significant: the higher the potential monetary reward, the better the participants recalled the answer 1 day later (Reward (None/Low/High): 52/55/57% correct; Wald χ^2^ = 11.656; *p* = 0.003), which is also consistent with earlier findings (Adcock et al. 2006). However, interaction between monetary reward and curiosity on memory was not significant (Low Curiosity combined with three levels of reward (None/Low/High): 38/43/46% correct; Middle curiosity combined with three levels of reward (None/Low/High): 51/54/56% correct; High curiosity combined with three levels of reward (None/Low/High): 66/67/68% correct; Wald χ^2^ = 2.79, *p* = 0.59). The results are summarized in Fig. 2.

**Fig. 3:**
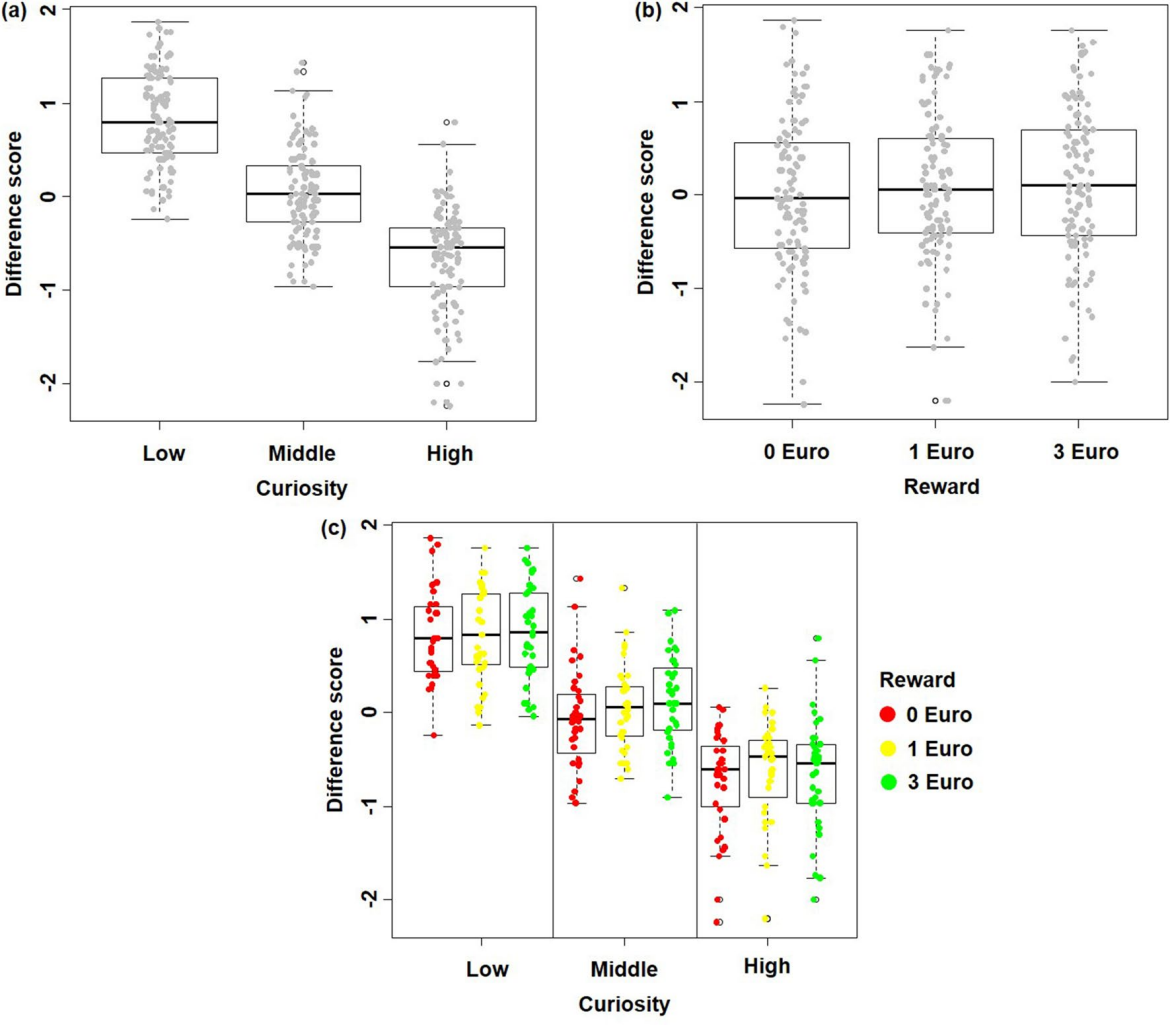
**a** The main effect of Curiosity: the higher the curiosity the better the memory performance. **b** The main effect of Monetary Reward: the higher the monetary reward the better the memory performance. **c** No significant interaction effect of Curiosity and Monetary Reward

There is a main effect of curiosity on prospective curiosity: the higher the level of curiosity, the less the participants want to know more about the topic as soon as their curiosity is satisfied (Curiosity (Low/Middle/High): 0.7663/0.0437/−0.6941; Wald χ^2^ = 579.486; *p* < 0.001)). The effect of reward on prospective curiosity was also significant: the higher the potential monetary reward on the trivia question, the more the participants want to know more about the topic in the future (Reward (None/Low/High): −0.0136/0.0358/0.0937; Wald χ^2^ = 6.816; *p* = 0.033). However, we did not find a significant interaction between reward and curiosity on memory (Wald χ^2^ = 1.48, *p* = 0.83). The results are summarized in Fig. 3.

### Brain activation associated with motivation during question presentation

We identified regions in which brain activity increased or decreased as a function of curiosity level and monetary reward during the trivia question phase.

**Fig. 4:**
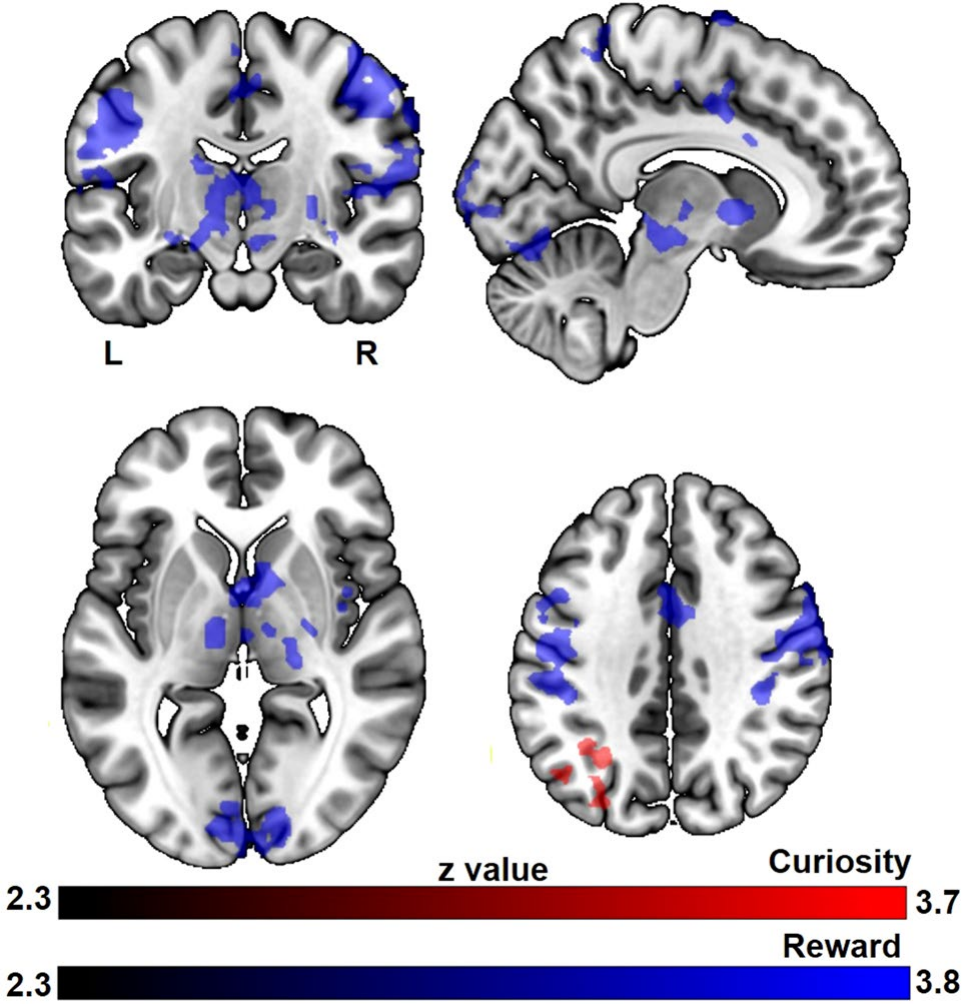
The main effects of curiosity and reward during trivia question presentation. All results are cluster-level corrected and thresholded (*p*_corr_ < 0.05 and *z* > 2.3)

For curiosity, activity in the middle temporal gyrus and inferior parietal lobule increased linearly with curiosity (Fig. 4, Table S1).

For monetary reward, activity in regions overlapping with well-known dopaminergic areas such as the pallidum and substantia nigra increased linearly with increasing monetary reward (Fig. 4, Table S2). Also, other brain regions known to be activated by reward anticipation such as the insula, anterior cingulate cortex (ACC), thalamus and pre- and postcentral gyrus showed activity that increased linearly with reward level.

### Interaction between motivation and memory on brain activity during answer presentation

**Fig. 5:**
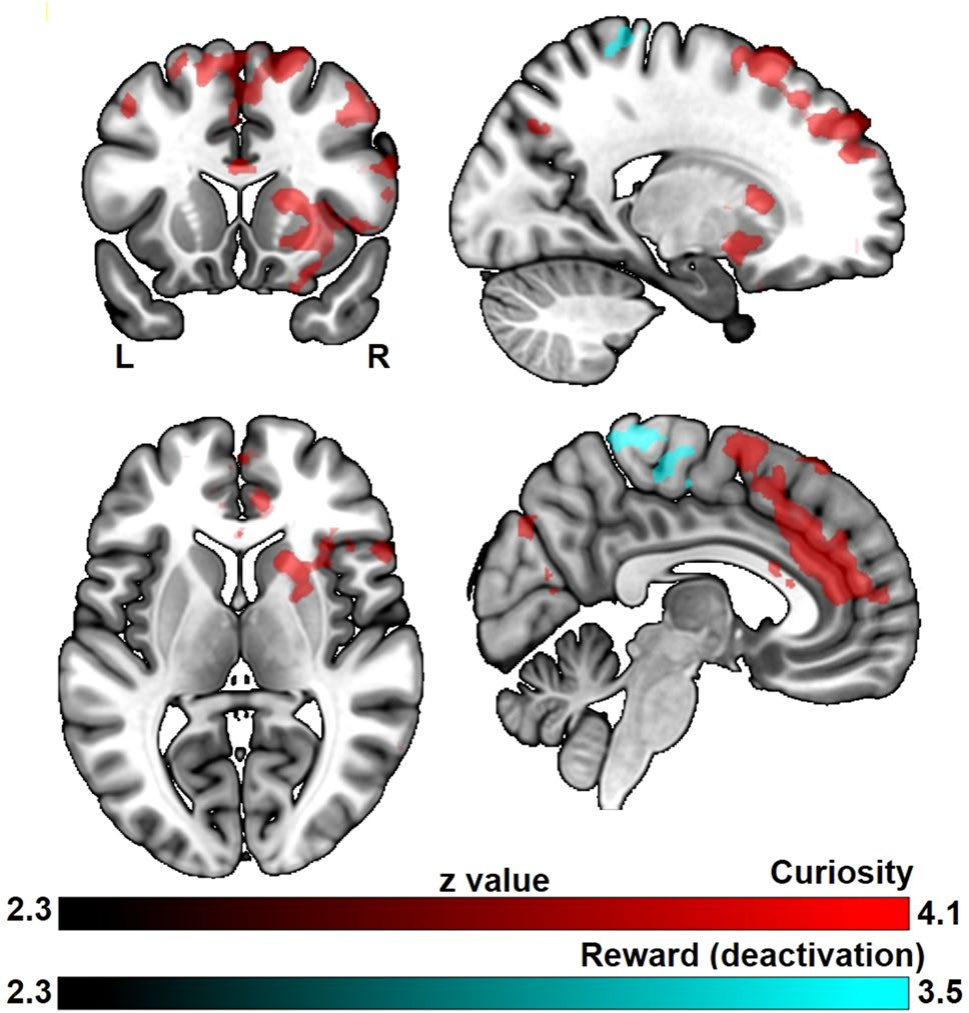
The subsequent memory effect modulated by curiosity and monetary reward. All results are cluster-level corrected and thresholded (*p*_corr_ < 0.05 and *z* > 2.3)

The subsequent memory effect was positively modulated by curiosity in dorsal and medial prefrontal cortices, inferior frontal gyrus, ACC, inferior parietal lobule, supramarginal gyrus, caudate nucleus and putamen (Fig. 5, Table S3). In contrast, deactivation in the precuneus and postcentral gyrus predicted the subsequent memory effect of trivia answer with higher reward level (Fig. 5, Table S4).

**Fig. 6:**
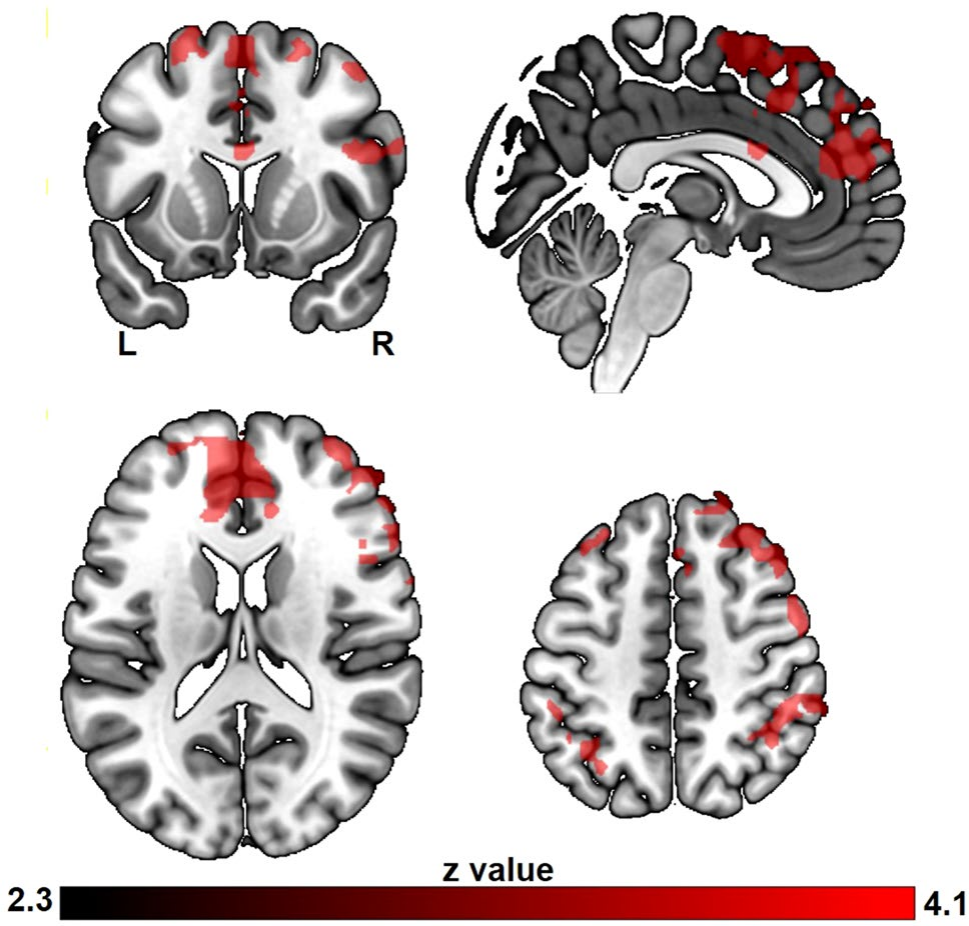
The differential effect between curiosity and reward on memory during trivia answer presentation. The colored regions were more active for the curiosity benefitted memory effect than the reward benefitted memory effect. All results are cluster-level corrected and thresholded (*p*_corr_ < 0.05 and *z* > 2.3)

To further investigate the relationship between monetary reward, curiosity and memory encoding, we contrasted the curiosity-modulated subsequent memory effect directly with the reward-modulated subsequent memory effect (contrast of two parametric modulations). Compared to the reward-modulated subsequent memory effect, the curiosity-modulated subsequent memory effect showed increased activation in prefrontal cortices extending to ACC, inferior parietal lobule and middle temporal gyrus (Fig. 6, Table S5). It should be noted that the topography of this comparison is very similar to the activation of the curiosity based subsequent memory effect, which is also located within this fronto-parietal network, suggesting that the fronto-parietal network might contain the main regions where curiosity interacts with memory formation. In contrast, no significant brain activation was found for the reverse contrast, which aimed to identify brain regions showing increased activation for the reward-modulated subsequent memory effect compared to the curiosity-modulated subsequent memory effect.

### Explorative analysis

**Fig. 7:**
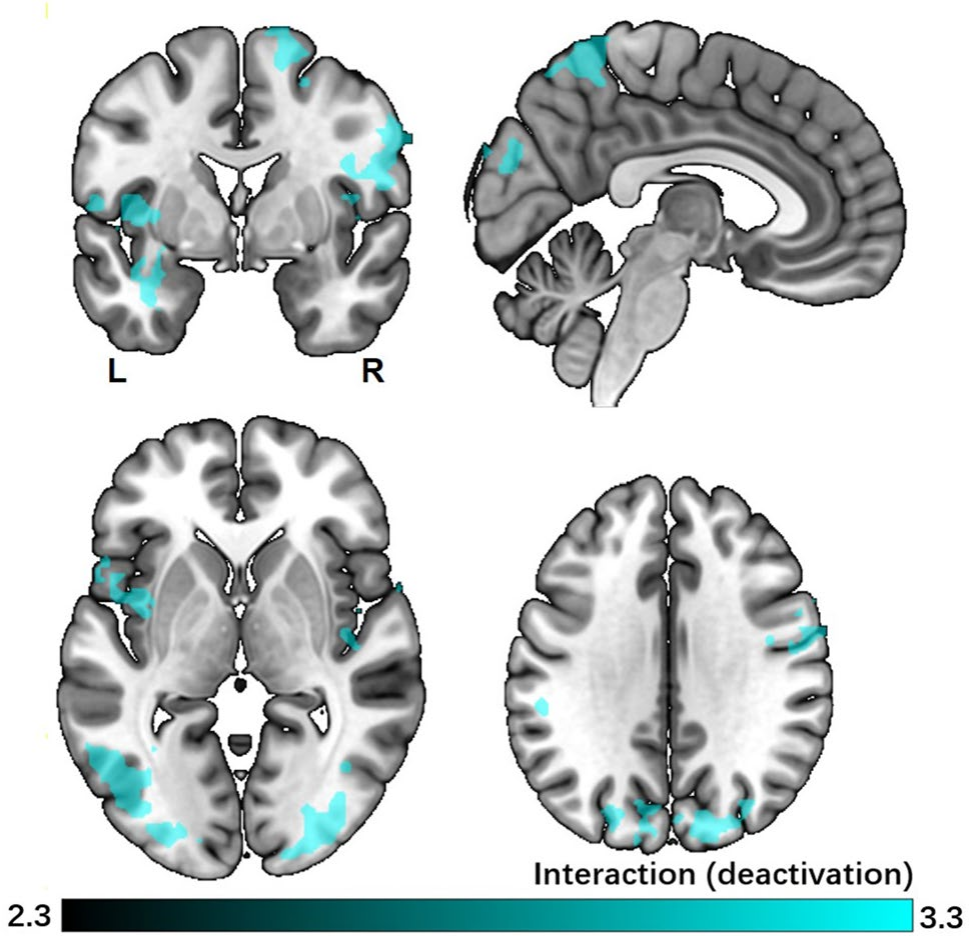
The interaction effect between curiosity and reward on subsequent memory effect. The colored regions were more active for the highly motivated (high curiosity and high monetary reward) questions in which the associated answers were subsequently forgotten than subsequently remembered. All results are cluster-level corrected and thresholded (*p*_corr_ < 0.05 and *z* > 2.3)

Though we did not find any interaction effect in behavioral performance, we nevertheless explored the interaction effect in brain activity during the question phase between monetary reward and curiosity. The analysis of the interaction effect between monetary reward and curiosity in subsequent memory showed that in highly motivated trials, there is deactivation in an occipital region of visual cortex, (supplementary) motor cortex, posterior insula and auditory cortex for the highly motivated (high curiosity and high monetary reward) questions in which the associated answers were subsequently remembered (Fig. 7).

When prior knowledge is controlled, the brain activation pattern is similar to the above results only with more restricted activation (see *fMRI results* part and Figs. S2–S5 in supplementary material).

### ROI results

**Fig. 8:**
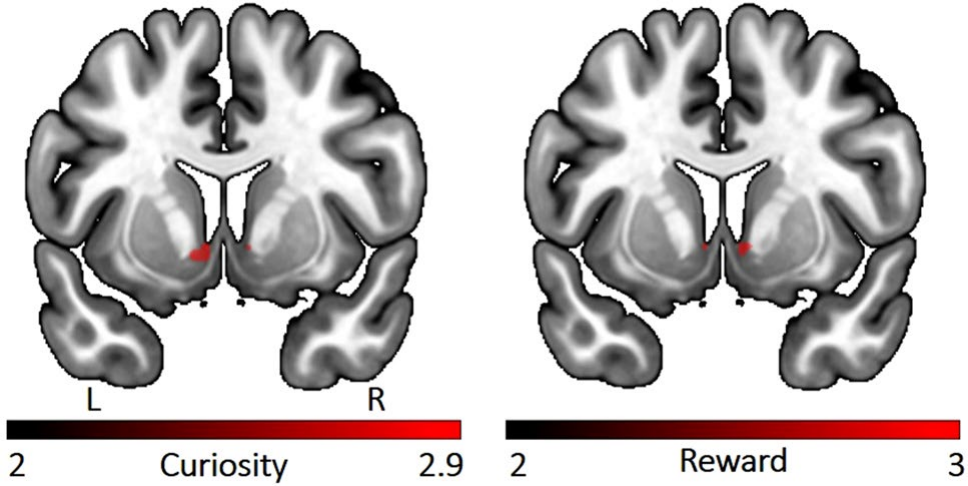
Left: NAcc activity modulated by curiosity level of the trivia question whose answer was later remembered. Right: NAcc activity modulated by monetary reward level of the trivia question whose answer was later remembered. Results are small volume corrected and thresholded at the voxel level (*p*_corr_ < 0.05)

The ROI analysis revealed that the NAcc activity was linearly increased with curiosity level for the trivia question whose answer was later remembered (Fig. 8 Left). Furthermore, this same association was also found between NAcc and reward level for the trivia question whose answer was later remembered (Fig. 8 Right). However, we did not find any significant effect in the hippocampus ROI.

## Discussion

The present results reveal the neural underpinnings of extrinsic and intrinsic motivation on memory formation obtained during the same task. We measured brain activity during memory encoding which was modulated by intrinsic (monetary reward) and extrinsic (curiosity) motivation during an intentional learning task. The brain activation during the trivia question phase and answer phase (see Fig. 1) was analyzed separately. Curiosity is supposed to be aroused and monetary reward anticipated during the display of the trivia question (with associated monetary reward). Curiosity is relieved when the trivia answer is presented.

Behaviorally, we found that both curiosity and monetary reward were associated with a significant increase in memory performance as tested 1 day later, which was consistent with previous findings when the effects of curiosity and reward were tested separately (Adcock et al. 2006; Gruber et al. 2014; Kang et al. 2009; Wittmann et al. 2005). Furthermore, we found that there was no significant interaction between curiosity and reward, i.e., the effects of extrinsic monetary reward and intrinsic curiosity on memory encoding were in the current experiment additive: the higher the monetary reward and the higher the curiosity, the better the memory performance. This result is not in line with results of a study by Murayama and Kuhbandner (2011), which reported that extrinsic monetary reward improved memory performance only for uninteresting trivia answers (25/45% correct rate under no money and money conditions) but not for interesting trivia answers (55/59% correct rate under no money and money conditions). While descriptively and numerically such a pattern is also observable on top of effects of both reward and curiosity in our data, i.e., 38/43/46% correct rate for low curiosity trivia answers under no/low/high money reward level; 51/54/56% correct rate for middle curiosity trivia answers under three money reward levels; 66/67/68% correct rate for high curiosity trivia answers under three money reward levels, this interaction does not reach significance, thus might be the size of effect too small to detect given the study samples’ power. The absence of a significant interaction effect in our sample might be due to the different paradigm we used in the current study. For example, Murayama and Kuhbandner (2011) used incidental learning while we instructed participants to memorize answers intentionally for a subsequent test. Incidental and intentional learning rely on different encoding operations, as incidental learning is relatively unaffected by encoding strategies, which normally facilitate intentional learning (Craik and Tulving 1975; Graf and Mandler 1984; Greene 1986). In addition, no performance-based monetary reward was provided in Murayama and Kuhbandner (2011)’s study; while, our participants received the associated monetary reward of 16 randomly chosen trivia questions if they recalled the corresponding answers correctly. Thus, an interactive modulation of memory formation by intrinsic and extrinsic motivation might only occur under specific conditions that were not tested here.

### Differential activation patterns of intrinsic and extrinsic motivation

Investigating the BOLD response associated with curiosity induction during the trivia question phase revealed activation in posterior middle temporal gyrus and inferior parietal lobule extended into the angular gyrus. These regions have been demonstrated to be crucially involved in semantic control (Davey et al. 2015, 2016; Whitney et al. 2010). Semantic control “not only requires us to access our storage of semantic facts but also to manipulate this information such that task-relevant aspects of meaning are brought to the fore” (Whitney et al. 2010). As such, semantic control implemented by these regions might partially underlie curiosity, in which participants need to access their stored knowledge and compare the trivia with the semantic knowledge they already have. Furthermore, activation in inferior parietal lobule covariates with curiosity in our study, which is also consistent with prior studies in which the inferior parietal lobule has been implicated in processing outcome uncertainty (Huettel et al. 2005; van Lieshout et al. 2018). Since we only chose trivia questions to which the participants themselves indicated they did not know the answer, our participants likely also were uncertain about the trivia information.

Independent of curiosity, anticipation of higher monetary reward was associated with activity in regions overlapping with well-known dopaminergic areas including pallidum and extended regions of substantia nigra, as well as other brain regions known to be activated by reward anticipation such as insula, ACC, thalamus and pre- and postcentral gyrus. The result from ROI further confirmed the corresponding parametric relationship between NAcc activation and monetary reward magnitude. This activation pattern of reward anticipation was consistent with previous monetary reward studies (Adcock et al. 2006; Wittmann et al. 2005).

Results from the question phase suggest that the memory benefit of extrinsic reward depends on its properties (monetary reward magnitude as in the present study), and that anticipation to get this reward is implemented in striatal regions. In contrast, the value of new information (curiosity level) potentially more strongly depends on semantic and epistemic factors to establish the intrinsic relevance of information. Curiosity is then induced through individuals’ awareness of the discrepancy between their current informational and goal uncertainty states (Gottlieb et al. 2013). This uncertainty-induced curiosity appears to be associated with parietal processes (Huettel et al. 2005; van Lieshout et al. 2018). Here, we operationalized monetary reward anticipation and curiosity by presenting individually rated trivia questions combined with potential monetary reward. Our results suggest that extrinsic and intrinsic motivation are implemented by different neural systems.

Interestingly, we found that the more the participant is curious about the trivia question during the *Screening* phase, the more their desire to know more about the topic is reduced once their curiosity is satisfied by seeing the answer to the trivia question during the *Study* phase. This could be interpreted as curiosity relief, which might be rewarding by the fulfillment of the information gap. Indeed, previous studies have revealed that this curiosity relief induces neural responses in the reward-related regions of the striatum (Jepma et al. 2012; Ligneul et al. 2018; van Lieshout et al. 2018). At the same time, the higher the potential monetary reward they could get on the trivia question, the more the participant wants to know more about the topic in the future. This result echoed with Eisenberger et al. (1999) study, in which compared to the non-reward group, participants who were offered monetary reward during a learning phase if they meet a certain standard on a task had higher levels of intrinsic motivation (as indicated by rating of task enjoyment and free time on task). In our experimental design, participants only receive monetary reward after the *Test* phase. In such a situation, delayed monetary reward may boost their prospective curiosity. This interpretation is consistent with the situational interest proposed by Hidi and Harackiewicz (2000) in which extrinsic motivation can become situational interest in a certain context and be maintained over time. Such conclusion could be potentially relevant for educational policy where extrinsic motivation like delayed reward delivery might benefit students who lack intrinsic motivation for academic activities at the beginning.

Though we did not find any interaction effect in behavioral performance, we nevertheless explored the specific activation pattern that pertains in the high reward combined with high curiosity condition, i.e., the facilitation effect of high extrinsic and intrinsic motivation on memory. The results showed that there is deactivation in occipital visual region, (supplementary) motor cortex, posterior insula and auditory cortex for the subsequent memory effect superadditive to the main effects of curiosity and reward. Deactivation seems to occur in several primary sensory brain areas. A speculative interpretation might be that individuals internalize strongly in this condition, potentially to concentrate on the material. This internalization decreases processing external and potentially distracting sensory input, thus enhancing memory encoding to the following associated answers.

### Neural mechanisms underlying the Effect of Intrinsic and Extrinsic Motivation on Memory

Next to activation in other brain regions, we found that activity in mesolimbic regions including putamen and caudate nucleus increased with curiosity for subsequently remembered trivia answers compared to forgotten ones. This result is consistent with a previous study on relief of perceptual curiosity (Jepma et al. 2012) and echoes with the idea that relief of curiosity is rewarding and promotes learning (Berlyne 1954; Loewenstein 1994). When the trivia answer is presented, the uncertainty about that information is resolved, which in turn can be rewarding and thus promotes learning. Besides reward-related brain regions, activity in a set of regions overlapping with the fronto-parietal attention network (Corbetta and Shulman 2002; Smith et al. 2009) was linearly associated with the curiosity level of subsequently remembered trivia answers. Evidence from eye-tracking data has previously shown that information of a higher curiosity level draws more attention (Baranes et al. 2015; Gottlieb et al. 2013; Kang et al. 2009). This endogenously biased attention to information helps individuals to learn, because it biases sampling from an information-rich environment, a necessity for “any limited-capacity organism that can sense much more information than it can fully process” (Gottlieb and Oudeyer 2018). Therefore, for the successfully remembered trivia answers, curiosity-driven activity in the ventral striatal reward network appears to work cooperatively with the fronto-parietal attention network, while enhancing memory formation.

Unlike the curiosity-modulated subsequent memory effect, the monetary reward-modulated subsequent memory effect was associated with decreased activity in posterior midline structures, i.e., greater activity was found during encoding of subsequently forgotten trivia items compared to subsequently remembered ones (negative subsequent memory effect) (Kim 2011; Weis et al. 2004). This deactivation partially overlaps with findings in previous reports (Daselaar et al. 2004; Otten and Rugg 2001a; Shrager et al. 2008; Wagner and Davachi 2001), where such effects were explained by diversion of neurocognitive resources away from effective encoding and into goal-inappropriate processes. (Wagner and Davachi 2001). Reward can gain automatic attentional priority regardless of action value in parietal regions (Gottlieb et al. 2014; Peck et al. 2009); the deactivation in the precuneus and postcentral gyrus might, therefore, suggest that participants need to suppress reward-biased, but memory-irrelevant processing, i.e., suppress their attention to reward itself rather than the to-be-memorized information to improve their memory performance.

We did not find evidence for an MTL involvement in monetary reward- or curiosity-modulated subsequent memory effects. However, hippocampal activity was found to be modulated by reward when only focusing on subsequently remembered items (see Table S2). This analysis approach and result is consistent with the report of Shrager et al. (2008) in which they showed that MTL activity increased with memory strength of subsequently recognized items only (Shrager et al. 2008). Many reasons might have limited our capacity to reveal a medial temporal subsequent memory effect (Henson 2005; Otten and Rugg 2001b). One possibility is that there was robust MTL activity unrelated to the task or similar for subsequently forgotten and remembered items (Wagner and Davachi 2001). In addition, the MTL subsequent memory effect is modulated by the stimuli to be memorized: the MTL subsequent memory effect is thought to be rather robust when encoding pictorial material but weaker when encoding verbal material (Kim 2011). Another possibility is that the difficulty of our memory task is not optimal to produce the reward-related enhancement of memory. Shigemune et al. (2017) found that monetary reward-related activation of the MTL was greater during the retrieval of memories with high difficulty (encoded with shallow strategy) than those with low difficulty (encoded with deep strategy). The average memory performance was about 54% correct in our study, indicating the memory task might not be difficult enough to activate the MTL.

In our results, increasing ventral striatal activity was observed for both increasing levels of monetary reward and curiosity, yet those at different times. Monetary reward modulated reward-related brain areas when seeing the question (and the indication of the potential reward) and curiosity modulated these areas when seeing the answer to the question. Thus, one could speculate that the monetary reward-related activity is more anticipatory in nature (Oldham et al. 2018); while, the reward-related brain activity elicited by curiosity is rather related to resolution or outcome. Furthermore, the direct comparison of the curiosity-modulated subsequent memory effect and the monetary reward-modulated subsequent memory effect showed that these fronto-parietal regions were more strongly activated in the context of curiosity-modulated memory formation. However, the reverse comparison revealed no significant activation or deactivation. This result echoed to some extent the behavioral performance that memory seems more strongly benefitted by intrinsic curiosity than extrinsic reward. More studies are needed to test the different time-course and magnitude effect of extrinsic and intrinsic motivation on performance. Furthermore, it is worthwhile to mention that though both intrinsic and extrinsic motivation improve memory performance after one-day delay, they might have differential influences on longer-term memory. Previous studies suggest that intrinsic motivation promotes more elaborative learning by more active engagement and thus, it will also last longer beyond the point of being rewarded, while extrinsic motivation promotes more superficial rote learning that might undermine future knowledge acquisition (Benware and Deci 1984; Kuhbandner et al. 2016; Vansteenkiste et al. 2004).

## Conclusions

By operationalizing extrinsic motivation as potential monetary reward and intrinsic motivation as curiosity, the present study provides the first demonstration of the neural correlates of how intrinsic motivation in the context of extrinsic motivation influences memory. Our behavioral results replicate recent findings that extrinsic motivation and intrinsic motivation separately promote memory formation. Neuroimaging data showed different neural mechanisms underlying extrinsic and intrinsic motivation and involvement during memory formation. Importantly, curiosity activated inferior parietal lobule and relief of curiosity activated brain reward regions. This is consistent with the theory that curiosity arises from uncertainty and that termination of this uncertainty is rewarding and promotes learning. Monetary reward enhances learning by focusing to suppress irrelevant information during encoding, which might be triggered by reward anticipation. Together, these findings suggest that both intrinsic and extrinsic motivation can benefit memory as tested here, although by different mechanisms and, thus, in an additive way.

## Electronic supplementary material

Below is the link to the electronic supplementary material.

Supplementary material 1 (DOCX 2776 kb)

## References

[CR1] Adcock RA, Thangavel A, Whitfield-Gabrieli S, Knutson B, Gabrieli JD (2006). Reward motivated learning: mesolimbic activation precedes memory formation. Neuron.

[CR2] Andersson JL, Jenkinson M, Smith S (2007) Non-linear registration, aka Spatial normalisation FMRIB technical report TR07JA2. FMRIB Analysis Group of the University of Oxford, 2: 1–21

[CR3] Baranes A, Oudeyer PY, Gottlieb J (2015). Eye movements reveal epistemic curiosity in human observers. Vision Res.

[CR4] Benware CA, Deci EL (1984). Quality of learning with an active versus passive motivational set. Am Educ Res J.

[CR5] Berlyne DE (1954). A theory of human curiosity. Br J Psychol.

[CR6] Bunzeck N, Doeller CF, Dolan RJ, Duzel E (2012). Contextual interaction between novelty and reward processing within the mesolimbic system. Hum Brain Mapp.

[CR7] Cerasoli CP, Nicklin JM, Ford MT (2014). Intrinsic motivation and extrinsic incentives jointly predict performance: a 40-year meta-analysis. Psychol Bull.

[CR8] Corbetta M, Shulman GL (2002). Control of goal-directed and stimulus-driven attention in the brain. Nat Rev Neurosci.

[CR9] Craik FI, Tulving E (1975). Depth of processing and the retention of words in episodic memory. J Exp Psychol Gen.

[CR10] Daselaar SM, Prince SE, Cabeza R (2004). When less means more: deactivations during encoding that predict subsequent memory. Neuroimage.

[CR11] Davey J, Cornelissen PL, Thompson HE, Sonkusare S, Hallam G, Smallwood J, Jefferies E (2015). Automatic and controlled semantic retrieval: TMS reveals distinct contributions of posterior middle temporal gyrus and angular gyrus. J Neurosci.

[CR12] Davey J (2016). Exploring the role of the posterior middle temporal gyrus in semantic cognition: integration of anterior temporal lobe with executive processes. NeuroImage.

[CR13] Deci EL, Koestner R, Ryan RM (1999). A meta-analytic review of experiments examining the effects of extrinsic rewards on intrinsic motivation. Psychol Bull.

[CR14] Desikan RS, Ségonne F, Fischl B, Quinn BT, Dickerson BC, Blacker D (2006). An automated labeling system for subdividing the human cerebral cortex on MRI scans into gyral based regions of interest. Neuroimage.

[CR15] Eisenberger R, Rhoades L, Cameron J (1999). Does pay for performance increase or decrease perceived self-determination and intrinsic motivation?. J Pers Soc Psychol.

[CR16] Gottlieb J, Oudeyer PY (2018). Towards a neuroscience of active sampling and curiosity. Nat Rev Neurosci.

[CR17] Gottlieb J, Oudeyer PY, Lopes M, Baranes A (2013). Information-seeking, curiosity, and attention: computational and neural mechanisms. Trends Cogn Sci.

[CR18] Gottlieb J, Hayhoe M, Hikosaka O, Rangel A (2014). Attention, reward, and information seeking. J Neurosci.

[CR19] Graf P, Mandler G (1984). Activation makes words more accessible, but not necessarily more retrievable. J Verbal Learning Verbal Behav.

[CR20] Greene RL (1986). Word stems as cues in recall and completion tasks. Q J Exp Psychol.

[CR21] Gruber MJ, Gelman BD, Ranganath C (2014). States of curiosity modulate hippocampus-dependent learning via the dopaminergic circuit. Neuron.

[CR22] Henson R (2005). A mini-review of fMRI studies of human medial temporal lobe activity associated with recognition memory. Q J Exp Psychol B.

[CR23] Hidi S, Harackiewicz JM (2000). Motivating the academically unmotivated: a critical issue for the 21st century. Rev Educ Res.

[CR24] Huettel SA, Song AW, McCarthy G (2005). Decisions under uncertainty: probabilistic context influences activation of prefrontal and parietal cortices. J Neurosci.

[CR25] Jenkinson M, Bannister P, Brady M, Smith S (2002). Improved optimization for the robust and accurate linear registration and motion correction of brain images. Neuroimage.

[CR26] Jenkinson M, Beckmann CF, Behrens TE, Woolrich MW, Smith SM (2012). Fsl. Neuroimage.

[CR27] Jepma M, Verdonschot RG, Van Steenbergen H, Rombouts SA, Nieuwenhuis S (2012). Neural mechanisms underlying the induction and relief of perceptual curiosity. Front Behav Neurosci.

[CR28] Kahn I, Shohamy D (2012). Instrinsic connectivity between the hippocampus, nucleu accumbens, and ventral tegmental area in humans. Hippocampus.

[CR29] Kang MJ, Hsu M, Krajbich IM, Loewenstein G, McClure SM, Wang JTY, Camerer CF (2009). The wick in the candle of learning: epistemic curiosity activates reward circuitry and enhances memory. Psychol Sci.

[CR30] Kidd C, Hayden BY (2015). The psychology and neuroscience of curiosity. Neuron.

[CR31] Kim H (2011). Neural activity that predicts subsequent memory and forgetting: a meta-analysis of 74 fMRI studies. Neuroimage.

[CR32] Kuhbandner C, Aslan A, Emmerdinger K, Murayama K (2016) Providing extrinsic reward for test performance undermines long-term memory acquisition. Front Psychol 7: 79. http://doi.org/10.3389/fpsyg.2016.00079 10.3389/fpsyg.2016.00079 PMC4740952 26869978

[CR33] Ligneul R, Mermillod M, Morisseau T (2018). From relief to surprise: dual control of epistemic curiosity in the human brain. NeuroImage.

[CR34] Lisman JE, Grace AA (2005). The hippocampal-VTA loop: controlling the entry of information into long-term memory. Neuron.

[CR35] Loewenstein G (1994). The psychology of curiosity: a review and reinterpretation. Psychol Bull.

[CR36] Murayama K, Kuhbandner C (2011). Money enhances memory consolidation–But only for boring material. Cognition.

[CR37] Murayama K, Matsumoto M, Izuma K, Matsumoto K (2010). Neural basis of the undermining effect of monetary reward on intrinsic motivation. Proc Natl Acad Sci USA.

[CR38] Oldham S, Murawski C, Fornito A, Youssef G, Yücel M, Lorenzetti V (2018). The anticipation and outcome phases of reward and loss processing: a neuroimaging meta-analysis of the monetary incentive delay task. Hum Brain Mapp.

[CR39] Otten LJ, Rugg MD (2001). When more means less: neural activity related to unsuccessful memory encoding. Curr Biol.

[CR40] Otten LJ, Rugg MD (2001). Task-dependency of the neural correlates of episodic encoding as measured by fMRI. Cereb Cortex.

[CR41] Peck CJ, Jangraw DC, Suzuki M, Efem R, Gottlieb J (2009). Reward modulates attention independently of action value in posterior parietal cortex. J Neurosci.

[CR42] Pruim RH, Mennes M, van Rooij D, Llera A, Buitelaar JK, Beckmann CF (2015). ICA-AROMA: a robust ICA-based strategy for removing motion artifacts from fMRI data. Neuroimage.

[CR43] Rossato JI, Bevilaqua LR, Izquierdo I, Medina JH, Cammarota M (2009). Dopamine controls persistence of long-term memory storage. Science.

[CR44] Ryan RM, Deci EL (2000). Self-determination theory and the facilitation of intrinsic motivation, social development, and well-being. Am Psychol.

[CR45] Shigemune Y, Tsukiura T, Nouchi R, Kambara T, Kawashima R (2017). Neural mechanisms underlying the reward-related enhancement of motivation when remembering episodic memories with high difficulty. Hum Brain Mapp.

[CR46] Shrager Y, Kirwan CB, Squire LR (2008). Activity in both hippocampus and perirhinal cortex predicts the memory strength of subsequently remembered information. Neuron.

[CR47] Smith SM (2009). Correspondence of the brain’s functional architecture during activation and rest. Proc Natl Acad Sci U S A.

[CR48] Squire LR, Stark CE, Clark RE (2004). The medial temporal lobe. Annu Rev Neurosci.

[CR49] Stevens J (1996). Applied multivariate statistics for the social sciences.

[CR50] van Lieshout LL, Vandenbroucke AR, Müller NC, Cools R, de Lange FP (2018). Induction and relief of curiosity elicit parietal and frontal activity. J Neurosci.

[CR51] Vansteenkiste M, Simons J, Lens W, Sheldon KM, Deci EL (2004). Motivating learning, performance, and persistence: the synergistic effects of intrinsic goal contents and autonomy-supportive contexts. J Pers Soc Psychol.

[CR52] Wagner AD, Davachi L (2001). Cognitive neuroscience: forgetting of things past. Curr Biol.

[CR53] Weis S, Klaver P, Reul J, Elger CE, Fernández G (2004). Temporal and cerebellar brain regions that support both declarative memory formation and retrieval. Cereb Cortex.

[CR54] Whitney C, Kirk M, O’sullivan J, Lambon Ralph MA, Jefferies E (2010). The neural organization of semantic control: TMS evidence for a distributed network in left inferior frontal and posterior middle temporal gyrus. Cereb Cortex.

[CR55] Wittmann BC, Schott BH, Guderian S, Frey JU, Heinze HJ, Düzel E (2005). Reward-related FMRI activation of dopaminergic midbrain is associated with enhanced hippocampus-dependent long-term memory formation. Neuron.

[CR56] Worsley KJ (2001). Statistical analysis of activation images. Functional MRI: An introduction to methods.

[CR57] Zellner MR, Ranaldi R (2010). How conditioned stimuli acquire the ability to activate VTA dopamine cells: a proposed neurobiological component of reward-related learning. Neurosci Biobehav Rev.

