# Supplementary material for: The effect of intrinsic and extrinsic motivation on memory formation: insight from behavioral and imaging study
Source: Brain Struct Funct. 2020 Apr 29;225(5):1561–74. doi: 10.1007/s00429-020-02074-x (PMC7286947; doi:10.1007/s00429-020-02074-x)
Supplement: Supplementary file 1 — Supplementary material 1 (DOCX 2776 kb) [file 429_2020_2074_MOESM1_ESM.docx]

**Supplementary Materials**

**Behavioral result**

During the Screening phase (number of trivial questions: 770), there are correlations between these three rating scores. Curiosity has an inverted-U-shaped relationship with prior knowledge (Fig. S1a), which is consistent with prior study (Kang et al., 2009). Curiosity rating reaches its maximum when prior knowledge is around 3.5. Similarly, prospective curiosity (“would you like to know more about the topic”) has an inverted-U-shaped relationship with prior knowledge, with prospective curiosity rating reaching its maximum when prior knowledge was around 4 (Fig. S1c). Based on this, we chose the trivia questions for the Study phase with the knowledge level less than 4 to maximize the curiosity level. Curiosity level is linearly associated prospective curiosity (Fig. S1b, r = 0.917, p = 0.000).


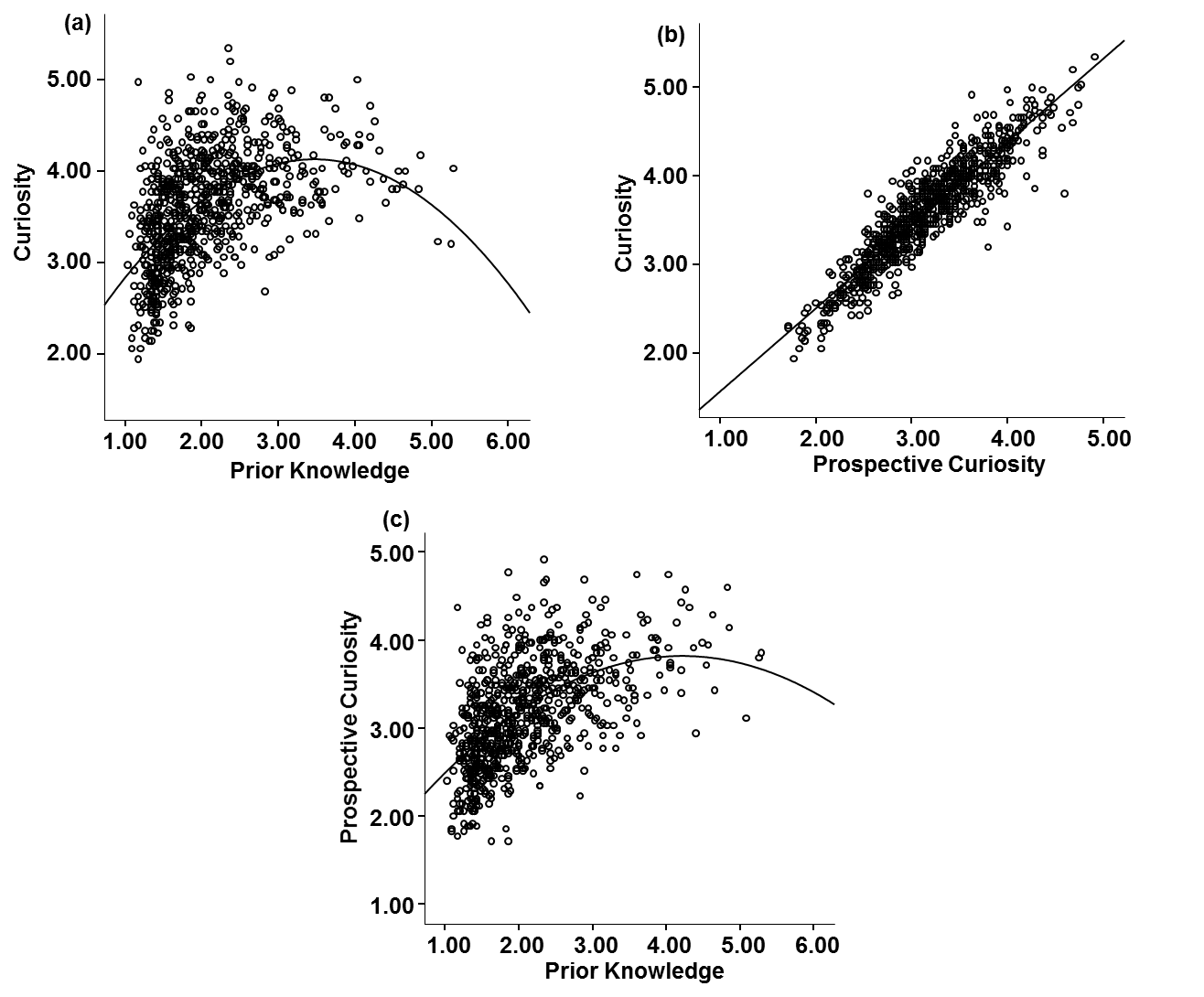


Fig S1. Scatter plot of (a) the relationship between prior knowledge and curiosity; (b) the relationship between curiosity and prospective curiosity; (c) the relationship between prior knowledge and prospective curiosity.

fMRI results

In our study design, prior knowledge is rated by subjective rating, which is a proxy of the familiarity to the question. Previous behavioral studies have indicated that familiarity and recall are functionally independent, but the extent and loci of their differential anatomical substrates are unclear (Diana et al., 2007; Rugg & Yonelinas, 2003). As prior knowledge is potentially related to both curiosity and reward, we did not include prior knowledge in our fMRI data analysis because it might ‘hijack’ the memory effect. Furthermore, the fMRI results showed that the activation pattern when prior knowledge is controlled is similar to our original report only with more restricted activation (See Fig S2-S5).


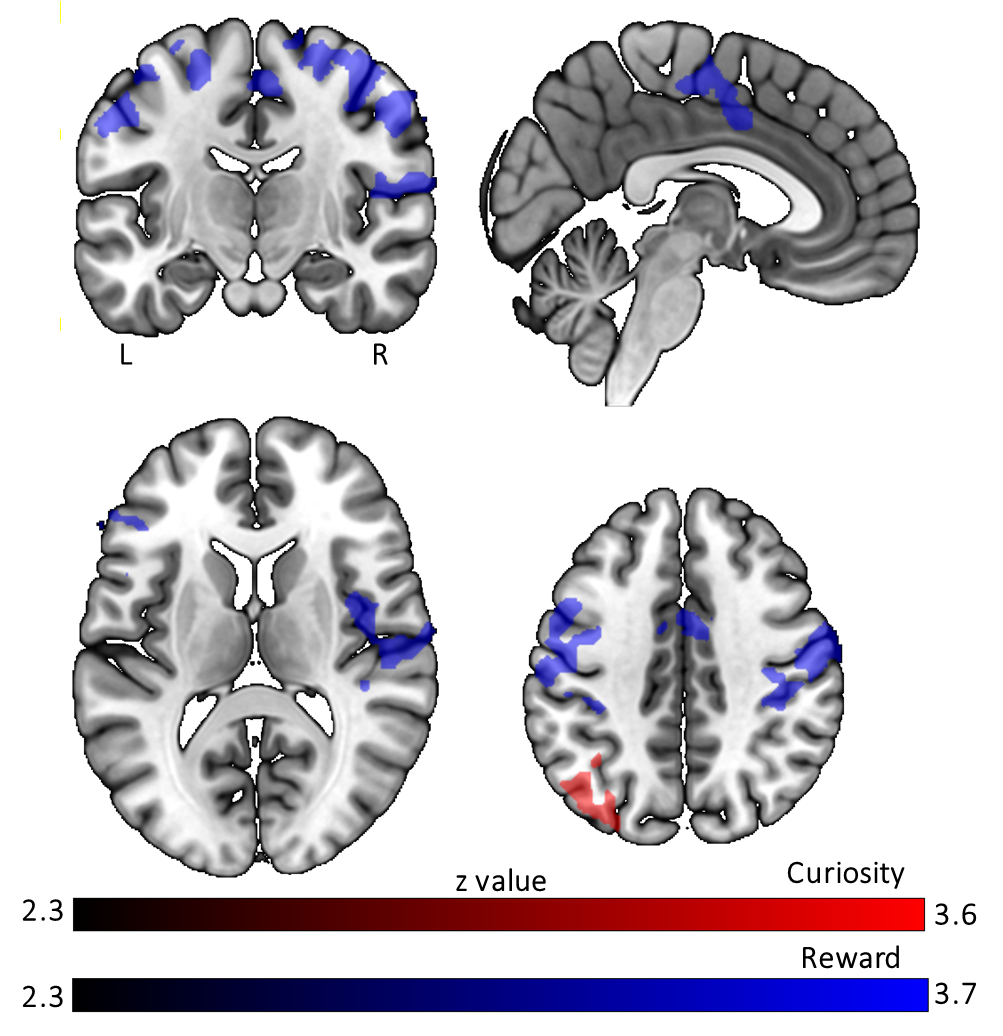


Fig S2. The main effect of curiosity and reward during trivia question presentation when the prior knowledge is controlled. All results are cluster-level corrected and thresholded (pcorr < 0.05 and z > 2.3).


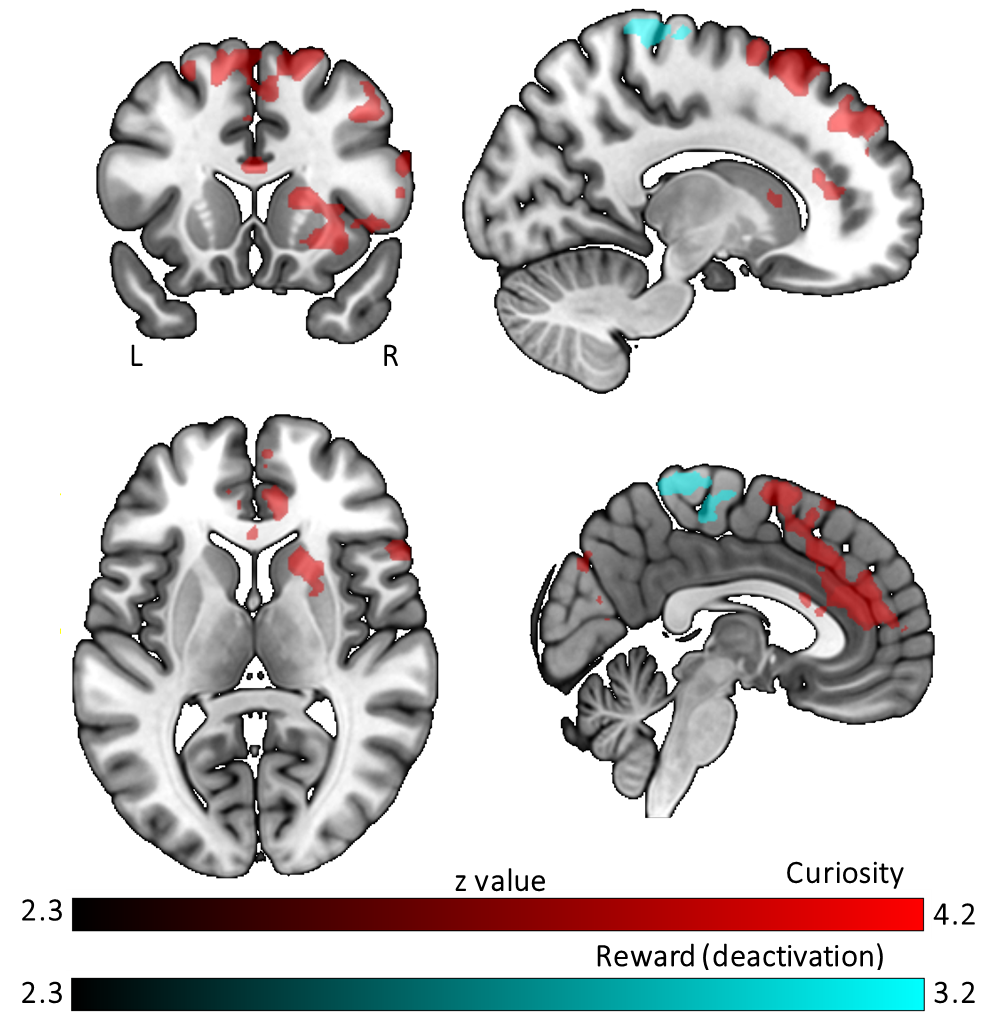


Fig S3. The subsequent memory effect modulated by curiosity and monetary reward when the prior knowledge is controlled. All results are cluster-level corrected and thresholded (pcorr < 0.05 and z > 2.3).


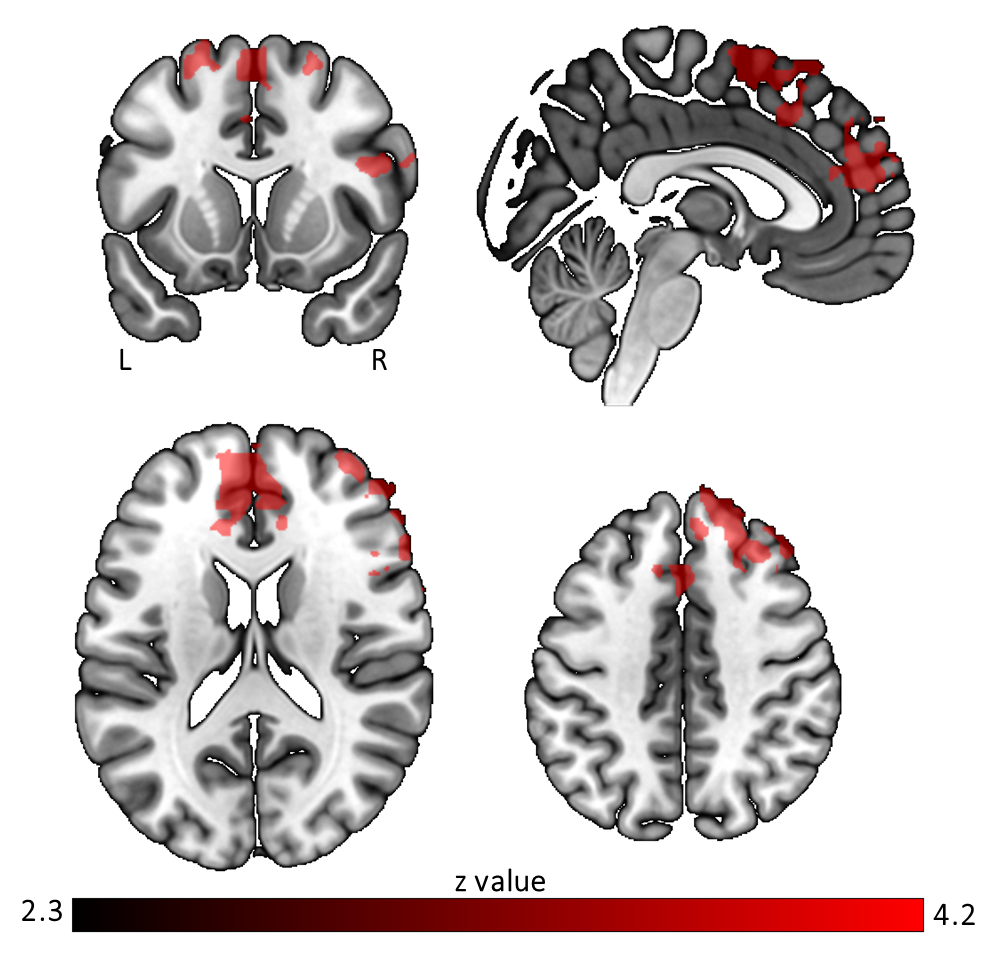


Fig S4. The differential effect between curiosity and reward on memory during trivia fact presentation when the prior knowledge is controlled. The colored regions were more active for the curiosity benefitted memory effect than the reward benefitted memory effect. All results are cluster-level corrected and thresholded (pcorr < 0.05 and z > 2.3).


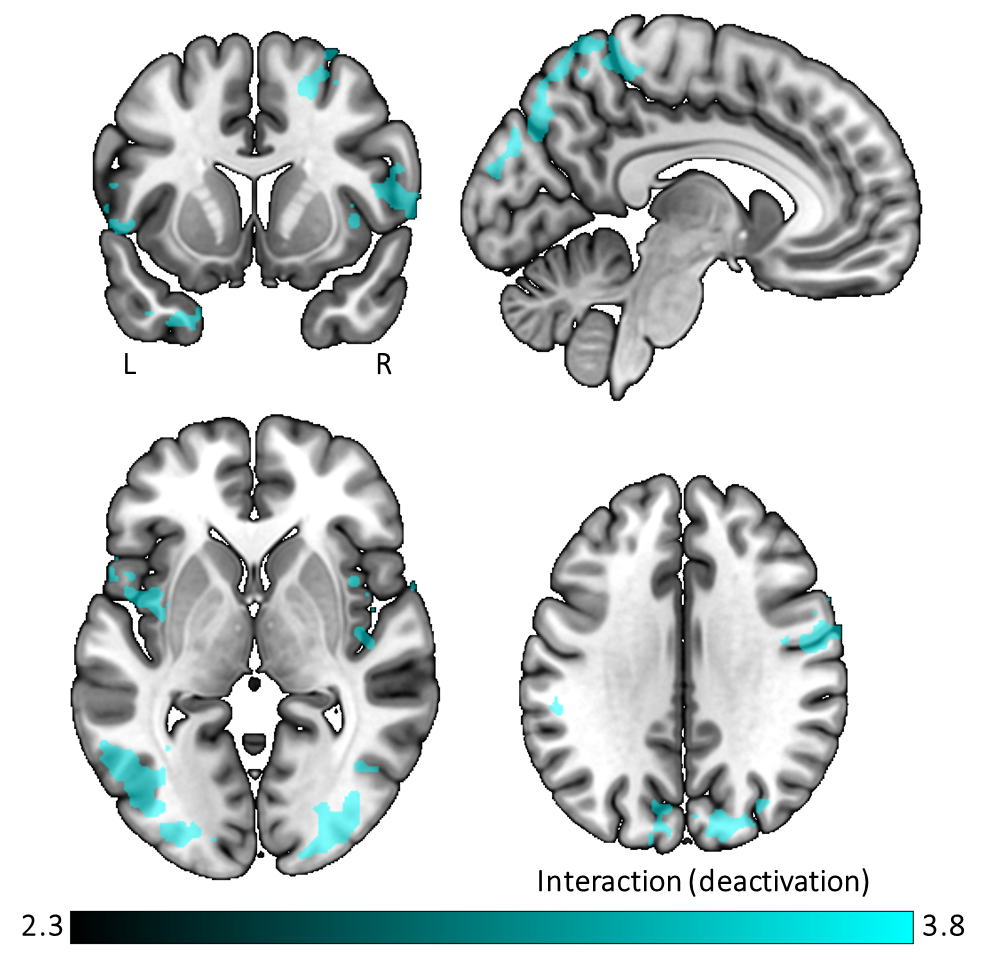


Fig S5. The interaction effect between curiosity and reward on subsequent memory effect when the prior knowledge is controlled. The colored regions were more active for the highly motivated (high curiosity and high monetary reward) questions in which the associated facts were subsequently forgotten than subsequently remembered. All results are cluster-level corrected and thresholded (pcorr < 0.05 and z > 2.3).

Table S1. GLM modulation of curiosity during trivia question presentation on brain activation.

|  |  |  |  | MNI coordinates | | |  |
| --- | --- | --- | --- | --- | --- | --- | --- |
| Area | Hemisphere | Cluster | Cluster extent | x | y | z | Peak voxel z-value |
| Middle Temporal Gyrus | L | 1 | 427 | -62 | -48 | -12 | 3.49 |
|  | L | 1 |  | -62 | -44 | -10 | 3.15 |
|  | L | 1 |  | -56 | -34 | -6 | 3.13 |
|  | L | 1 |  | -58 | -50 | -4 | 3.12 |
|  | L | 1 |  | -50 | -32 | -8 | 3.12 |
|  | L | 1 |  | -54 | -20 | -8 | 2.95 |
|  | L | 1 |  | -66 | -34 | -6 | 2.91 |
| Inferior Temporal Gyrus | L | 1 |  | -58 | -48 | -18 | 2.91 |
|  | L | 1 |  | -66 | -42 | -20 | 2.6 |
|  | L | 1 |  | -50 | -32 | -8 | 3.12 |
| Inferior Parietal Lobule | L | 2 | 661 | -42 | -54 | 58 | 3.63 |
|  | L | 2 |  | -36 | -54 | 38 | 3.45 |
|  | L | 2 |  | -32 | -70 | 42 | 3.29 |
|  | L | 2 |  | -36 | -58 | 46 | 3.16 |
|  | L | 2 |  | -44 | -44 | 56 | 3.06 |
|  | L | 2 |  | -34 | -74 | 52 | 2.93 |
|  | L | 2 |  | -50 | -44 | 50 | 2.92 |
| Middle Occipital Gyrus | L | 2 |  | -28 | -62 | 38 | 3.35 |
|  | L | 2 |  | -30 | -72 | 40 | 3.2 |
|  | L | 2 |  | -28 | -76 | 40 | 3.09 |
| Angular Gyrus | L | 2 |  | -40 | -58 | 44 | 3.06 |

Note: cluster level corrected (FWE) at z>2.3 cluster defining threshold and corrected *p*<0.05, all local maxima are reported.

Table S2. GLM modulation of reward during question phase on brain activation.

|  |  |  |  | MNI coordinates | | |  |
| --- | --- | --- | --- | --- | --- | --- | --- |
| Area | Hemisphere | Cluster | Cluster extent | x | y | z | Peak voxel z-value |
| Calcarine Gyrus | L | 1 | 2 | -14 | -100 | -6 | 2.33 |
| Precentral Gyrus | L | 2 | 30 | -40 | -8 | 46 | 2.75 |
|  | L | 2 |  | -42 | 0 | 52 | 2.51 |
|  | L | 2 |  | -44 | -4 | 56 | 2.47 |
| Cuneus | L | 3 | 710 | -2 | -96 | 18 | 3.6 |
|  |  | 3 |  | 0 | -94 | 14 | 2.95 |
| Cuneus | R | 3 |  | 20 | -98 | 8 | 3.32 |
|  | R | 3 |  | 6 | -94 | 18 | 2.83 |
| Calcarine Gyrus | L | 3 |  | -6 | -94 | 0 | 3.59 |
|  |  | 3 |  | 0 | -100 | 12 | 3.17 |
|  | L | 3 |  | -10 | -88 | -4 | 3.05 |
|  |  | 3 |  | 0 | -96 | 2 | 2.86 |
| Calcarine Gyrus | R | 3 |  | 12 | -88 | 0 | 3.17 |
|  | R | 3 |  | 16 | -92 | 4 | 3.13 |
| Superior Occipital Gyrus | L | 3 |  | -12 | -94 | 6 | 2.9 |
| MCC | R | 4 | 935 | 2 | 2 | 42 | 3.86 |
|  | R | 4 |  | 4 | -2 | 42 | 3.7 |
|  | R | 4 |  | 8 | -4 | 44 | 3.26 |
| MCC |  | 4 |  | 0 | 8 | 34 | 3.82 |
| Posterior-Medial Frontal | R | 4 |  | 2 | -4 | 46 | 3.75 |
|  | R | 4 |  | 6 | 2 | 76 | 3.55 |
| Posterior-Medial Frontal |  | 4 |  | 0 | -8 | 62 | 3.59 |
|  | L | 4 |  | -2 | 0 | 66 | 3.34 |
|  | L | 4 |  | -4 | 2 | 54 | 3.3 |
|  |  | 4 |  | 0 | 2 | 72 | 3.01 |
| ACC | R | 4 |  | 6 | 12 | 28 | 3.31 |
| Fusiform Gyrus | R | 5 | 1042 | 40 | -38 | -28 | 3.34 |
| Cerebellar Vermis (7) |  | 5 |  | 0 | -76 | -22 | 3.31 |
| Cerebellar Vermis (6) | R | 5 |  | 2 | -74 | -18 | 3.24 |
| Lingual Gyrus | R | 5 |  | 8 | -72 | -12 | 3.23 |
| Lingual Gyrus | L | 5 |  | -4 | -74 | -8 | 3.14 |
| Cerebelum (V) | L | 5 |  | -14 | -54 | -26 | 3.22 |
| Cerebelum (VI) | L | 5 |  | -6 | -72 | -12 | 3.18 |
|  | R | 5 |  | -18 | -62 | -30 | 3.16 |
|  | R | 5 |  | 24 | -56 | -26 | 3.17 |
|  | R | 5 |  | 26 | -64 | -22 | 3.15 |
|  | R | 5 |  | 18 | -52 | -34 | 3.07 |
| Thalamus | L | 6 | 1406 | -10 | -16 | 2 | 4.29 |
|  | L | 6 |  | -4 | -16 | 12 | 3.47 |
|  | R | 6 |  | 2 | 0 | 2 | 3.67 |
|  | R | 6 |  | 2 | -16 | 12 | 3.45 |
|  | R | 6 |  | 20 | -26 | -4 | 3.41 |
|  | R | 6 |  | 2 | -18 | 6 | 3.59 |
| Substania Nigra/midbrain | R | 6 |  | 10 | -16 | -12 | 3.77 |
|  | R | 6 |  | 8 | -24 | -10 | 3.54 |
| Hippocampus | L | 6 |  | -18 | -20 | -12 | 3.6 |
| Pallidum | R | 6 |  | 10 | 4 | 0 | 3.57 |
|  | L | 6 |  | -14 | 4 | -2 | 3.47 |
| Postcentral Gyrus | L | 7 | 2065 | -52 | -16 | 32 | 4.26 |
|  | L | 7 |  | -50 | -10 | 42 | 3.9 |
|  | L | 7 |  | -48 | -8 | 38 | 3.87 |
|  | L | 7 |  | -58 | -18 | 14 | 3.53 |
|  | L | 7 |  | -60 | -16 | 32 | 3.48 |
| IFG (p. Opercularis) | L | 7 |  | -52 | 10 | 20 | 3.98 |
|  |  | 7 |  | -52 | 8 | 26 | 3.91 |
| Precentral Gyrus | L | 7 |  | -60 | -26 | 46 | 3.65 |
|  | L | 7 |  | -60 | 6 | 30 | 3.54 |
|  | L | 7 |  | -56 | 4 | 24 | 3.37 |
| Inferior Parietal Lobule | L | 7 |  | -44 | -32 | 38 | 3.43 |
| IFG (p. Opercularis) | R | 8 | 4275 | 62 | 14 | 26 | 4.35 |
| Rolandic Operculum | R | 8 |  | 66 | -6 | 8 | 3.97 |
|  |  | 8 |  | 62 | -18 | 14 | 3.64 |
| Postcentral Gyrus | R | 8 |  | 56 | -20 | 50 | 3.91 |
| Insula Lobe | R | 8 |  | 40 | 2 | 8 | 3.9 |
|  |  | 8 |  | 38 | 2 | 12 | 3.86 |
|  |  | 8 |  | 44 | -8 | 10 | 3.67 |
| Precentral Gyrus | R | 8 |  | 58 | -8 | 42 | 3.85 |
|  |  | 8 |  | 52 | -10 | 40 | 3.83 |
|  |  | 8 |  | 52 | -10 | 58 | 3.55 |
| Superior Frontal Gyrus | R | 8 |  | 36 | -4 | 62 | 3.63 |

Note: cluster level corrected (FWE) at z>2.3 cluster defining threshold and corrected *p*<0.05, all local maxima are reported.

Table S3. Curiosity modulated subsequent memory effect (remembered versus forgotten) on brain activation when trivia fact was presented.

|  |  |  |  | MNI coordinates | | |  |
| --- | --- | --- | --- | --- | --- | --- | --- |
| Area | Hemisphere | Cluster | Cluster extent | x | y | z | Peak voxel z-value |
| Middle Frontal Gyrus | R | 1 | 2 | 28 | 26 | 34 | 2.32 |
| Superior Medial Gyrus | L | 2 | 4 | -4 | 54 | 4 | 2.54 |
| Precuneus | L | 3 | 579 | -6 | -74 | 42 | 3.25 |
|  | L | 3 |  | -6 | -78 | 52 | 2.95 |
| Superior Occipital Gyrus | L | 3 |  | -22 | -68 | 38 | 3.07 |
|  | L | 3 |  | -16 | -70 | 38 | 2.87 |
| Cuneus | L | 3 |  | 0 | -72 | 20 | 3.07 |
|  | R | 3 |  | 8 | -78 | 38 | 2.97 |
|  | L | 3 |  | -2 | -78 | 34 | 2.9 |
|  | L | 3 |  | -14 | -72 | 36 | 2.87 |
|  | L | 3 |  | 2 | -76 | 34 | 2.87 |
|  | L | 3 |  | -2 | -74 | 24 | 2.84 |
| Middle Occipital Gyrus | L | 3 |  | -24 | -62 | 36 | 2.99 |
| Supramarginal Gyrus | R | 4 | 1134 | 62 | -46 | 32 | 4.13 |
|  | R | 4 |  | 64 | -42 | 48 | 3.24 |
|  | R | 4 |  | 54 | -46 | 42 | 3.2 |
|  | R | 4 |  | 64 | -32 | 40 | 3.09 |
| Inferior Parietal Lobule | R | 4 |  | 46 | -44 | 50 | 3.85 |
|  | R | 4 |  | 58 | -38 | 48 | 3.65 |
|  | R | 4 |  | 50 | -48 | 40 | 3.24 |
|  | R | 4 |  | 54 | -48 | 46 | 3.17 |
|  | R | 4 |  | 46 | -54 | 50 | 3.04 |
|  | R | 4 |  | 38 | -50 | 54 | 3.01 |
|  | R | 4 |  | 42 | -56 | 46 | 2.99 |
| Inferior Parietal Lobule | L | 5 | 1307 | -50 | -46 | 44 | 3.67 |
|  | L | 5 |  | -54 | -54 | 44 | 3.52 |
|  | L | 5 |  | -58 | -56 | 36 | 3.5 |
|  | L | 5 |  | -50 | -42 | 52 | 3.39 |
|  | L | 5 |  | -60 | -40 | 44 | 3.12 |
| Angular Gyrus | L | 5 |  | -58 | -54 | 42 | 3.59 |
|  | L | 5 |  | -46 | -54 | 34 | 3.49 |
| Supramarginal Gyrus | L | 5 |  | -64 | -44 | 30 | 3.58 |
|  | L | 5 |  | -64 | -50 | 36 | 3.22 |
|  | L | 5 |  | -58 | -44 | 50 | 3.1 |
|  | L | 5 |  | -58 | -44 | 34 | 3.36 |
| IFG (p. Triangular) | R | 6 | 1351 | 58 | 22 | 22 | 3.84 |
| Caudate Nucleus | R | 6 |  | 18 | 18 | 8 | 3.74 |
|  | R | 6 |  | 20 | 16 | 10 | 3.66 |
| Middle Orbital Gyrus | R | 6 |  | 44 | 50 | -4 | 3.66 |
|  | R | 6 |  | 24 | 54 | -12 | 3.29 |
| IFG (p. Orbitalis) | R | 6 |  | 20 | 16 | -26 | 3.59 |
| Insula lobe | R | 6 |  | 30 | 22 | -8 | 3.5 |
| Anterior Insula | R | 6 |  | 32 | 18 | -4 | 3.49 |
| Olfactory cortex | R | 6 |  | 26 | 12 | -18 | 3.37 |
| Putamen | R | 6 |  | 20 | 6 | -10 | 3.21 |
|  | R | 6 |  | 24 | 16 | -8 | 3.2 |
| ACC | R | 7 | 7698 | 6 | 44 | 12 | 4.19 |
|  | L | 7 |  | -8 | 42 | 14 | 3.85 |
| Superior Medial Gyrus | L | 7 |  | -8 | 40 | 30 | 4.16 |
|  | R | 7 |  | 16 | 24 | 64 | 3.82 |
| Middle Frontal Gyrus | R | 7 |  | 40 | 50 | 26 | 3.94 |
|  | R | 7 |  | 30 | 58 | 28 | 3.86 |
| Posterior-Medial Frontal | R | 7 |  | 14 | 22 | 60 | 3.9 |
|  | L | 7 |  | -6 | 24 | 66 | 3.88 |
|  | L | 7 |  | -4 | 24 | 60 | 3.8 |
| Frontal Pole | L | 7 |  | -28 | 64 | 20 | 3.89 |
| IFG (p. Triangularis) | R | 7 |  | 40 | 30 | 28 | 3.77 |

Note: cluster level corrected (FWE) at z>2.3 cluster defining threshold and corrected *p*<0.05, all local maxima are reported.

Table S4. Monetary reward modulated subsequent memory effect (remembered versus forgotten) on brain deactivation when trivia fact was presented.

|  |  |  |  | MNI coordinates | | |  |
| --- | --- | --- | --- | --- | --- | --- | --- |
| Area | Hemisphere | Cluster | Cluster extent | x | y | z | Peak voxel z-value |
| Paracentral Lobule | L | 1 | 25 | -6 | -30 | 54 | 2.67 |
|  | L | 1 |  | -2 | -26 | 52 | 2.62 |
|  |  | 1 |  | 0 | -34 | 56 | 2.59 |
| Postcentral Gyrus |  | 2 | 514 | 0 | -40 | 66 | 3.31 |
| Postcentral Gyrus | R | 2 |  | 14 | -30 | 76 | 3.16 |
|  | R | 2 |  | 20 | -38 | 74 | 2.99 |
|  | R | 2 |  | 22 | -42 | 68 | 2.89 |
| Posterior-Medial Frontal | R | 2 |  | 4 | -24 | 62 | 3.03 |
|  | L | 2 |  | -6 | -12 | 72 | 2.93 |
|  | R | 2 |  | 2 | -16 | 66 | 2.88 |
|  | R | 2 |  | 8 | -14 | 54 | 2.72 |
| Precuneus | R | 2 |  | 10 | -46 | 72 | 2.95 |
| Paracentral Lobule | L | 2 |  | -6 | -28 | 76 | 2.81 |
| Precentral Gyrus | R | 2 |  | 16 | -20 | 72 | 2.71 |

Note: cluster level corrected (FWE) at z>2.3 cluster defining threshold and corrected *p*<0.05, all local maxima are reported.

Table S5. During trivia fact presentation, Curiosity > Reward for subsequent memory effect.

|  |  |  |  | MNI coordinates | | |  |
| --- | --- | --- | --- | --- | --- | --- | --- |
| Area | Hemisphere | Cluster | Cluster extent | x | y | z | Peak voxel z-value |
| Angular gyrus | R | 1 | 368 | 42 | -60 | 54 | 2.45 |
| Superior parietal lobule | R |  |  | 54 | -36 | 58 | 2.54 |
| Inferior parietal lobule | R |  |  | 32 | -52 | 48 | 2.42 |
|  |  |  |  | 42 | -52 | 42 | 2.56 |
|  |  |  |  | 58 | -34 | 56 | 2.58 |
|  |  |  |  | 44 | -36 | 36 | 2.64 |
|  |  |  |  | 54 | -44 | 54 | 3.04 |
|  |  |  |  | 64 | -40 | 48 | 3.08 |
|  |  |  |  | 58 | -40 | 52 | 3.24 |
|  |  |  |  | 50 | -38 | 48 | 3.32 |
|  |  |  |  | 46 | -42 | 50 | 3.69 |
| Superior Parietal Lobule | L | 2 | 377 | -30 | -64 | 46 | 2.64 |
|  |  |  |  | -42 | -52 | 62 | 3.36 |
| Inferior parietal lobule | L |  |  | -38 | -56 | 54 | 2.68 |
|  |  |  |  | -44 | -52 | 46 | 2.74 |
|  |  |  |  | -48 | -42 | 50 | 2.78 |
|  |  |  |  | -50 | -38 | 40 | 2.87 |
|  |  |  |  | -50 | -38 | 46 | 2.88 |
|  |  |  |  | -46 | -46 | 58 | 3 |
|  |  |  |  | -36 | -56 | 48 | 3.1 |
| Middle occipital gyrus | L |  |  | -24 | -72 | 26 | 2.87 |
|  |  |  |  | -24 | -66 | 36 | 3.54 |
| Superior Temporal Gyrus | R | 3 | 396 | 58 | -30 | 8 | 2.62 |
|  |  |  |  | 64 | -42 | 12 | 2.67 |
|  |  |  |  | 60 | -34 | 6 | 2.78 |
|  |  |  |  | 56 | -10 | 2 | 2.83 |
|  |  |  |  | 68 | -30 | 2 | 2.98 |
|  |  |  |  | 62 | -28 | 0 | 3.25 |
| Middle temporal gyrus | R |  |  | 72 | -32 | -4 | 2.77 |
|  |  |  |  | 68 | -14 | -10 | 3.28 |
|  |  |  |  | 70 | -22 | -6 | 3.79 |
| Superior temporal gyrus | R |  |  | 60 | -18 | 0 | 3.32 |
| ACC | L | 4 | 6347 | -8 | 42 | 16 | 3.55 |
|  |  |  |  | -6 | 40 | 22 | 3.78 |
|  | R |  |  | 8 | 42 | 12 | 4 |
| Superior frontal gyrus | R |  |  | 14 | 26 | 60 | 3.59 |
|  |  |  |  | 10 | 34 | 58 | 3.64 |
|  |  |  |  | 10 | 40 | 40 | 3.67 |
| Middle frontal gyrus | R |  |  | 42 | 26 | 50 | 3.69 |
| IFG | R |  |  | 56 | 36 | 14 | 3.77 |
| Posterior-medial frontal gyrus | L |  |  | 0 | 8 | 62 | 3.66 |
|  |  |  |  | 0 | 4 | 64 | 3.89 |
|  |  |  |  | -6 | 8 | 58 | 4.24 |

Note: cluster level corrected (FWE) at z>2.3 cluster defining threshold and corrected *p*<0.05, all local maxima are reported.

Reference

Desikan RS, Ségonne F, Fischl B, Quinn BT, Dickerson BC, Blacker D et al (2006) An automated labeling system for subdividing the human cerebral cortex on MRI scans into gyral based regions of interest. Neuroimage 31: 968-980. http://doi.org/10.1016/j.neuroimage.2006.01.021

Diana RA, Yonelinas AP, Ranganath C (2007) Imaging recollection and familiarity in the medial temporal lobe: a three-component model. Trends Cogn Sci 11: 379-386. http://doi.org/10.1016/j.tics.2007.08.001

Kang MJ, Hsu M, Krajbich IM, Loewenstein G, McClure SM, Wang JTY, Camerer CF (2009) The wick in the candle of learning: Epistemic curiosity activates reward circuitry and enhances memory. Psychol Sci 20: 963-973. http://doi.org/10.1111/j.1467-9280.2009.02402.x

Rugg MD, Yonelinas AP (2003) Human recognition memory: a cognitive neuroscience perspective. Trends Cogn Sci 7: 313-319.
